# A proteomics study identifying interactors of the FSHD2 gene product SMCHD1 reveals RUVBL1-dependent DUX4 repression

**DOI:** 10.1038/s41598-021-03030-3

**Published:** 2021-12-08

**Authors:** Remko Goossens, Mara S. Tihaya, Anita van den Heuvel, Klorane Tabot-Ndip, Iris M. Willemsen, Stephen J. Tapscott, Román González-Prieto, Jer-Gung Chang, Alfred C. O. Vertegaal, Judit Balog, Silvère M. van der Maarel

**Affiliations:** 1 Department of Human Genetics, Leiden University Medical Center, Leiden, The Netherlands; 2 Human Biology Division, Fred Hutchinson Cancer Research Center, Seattle, USA; 3 Department of Cell and Chemical Biology, Leiden University Medical Center, Leiden, The Netherlands

**Keywords:** Chromatin, Epigenetics, Protein-protein interaction networks, Neuromuscular disease

## Abstract

Structural Maintenance of Chromosomes Hinge Domain Containing 1 (SMCHD1) is a chromatin repressor, which is mutated in > 95% of Facioscapulohumeral dystrophy (FSHD) type 2 cases. In FSHD2, SMCHD1 mutations ultimately result in the presence of the cleavage stage transcription factor DUX4 in muscle cells due to a failure in epigenetic repression of the D4Z4 macrosatellite repeat on chromosome 4q, which contains the *DUX4* locus. While binding of SMCHD1 to D4Z4 and its necessity to maintain a repressive D4Z4 chromatin structure in somatic cells are well documented, it is unclear how SMCHD1 is recruited to D4Z4, and how it exerts its repressive properties on chromatin. Here, we employ a quantitative proteomics approach to identify and characterize novel SMCHD1 interacting proteins, and assess their functionality in D4Z4 repression. We identify 28 robust SMCHD1 nuclear interactors, of which 12 are present in D4Z4 chromatin of myocytes. We demonstrate that loss of one of these SMCHD1 interacting proteins, RuvB-like 1 (RUVBL1), further derepresses DUX4 in FSHD myocytes. We also confirm the interaction of SMCHD1 with EZH inhibitory protein (EZHIP), a protein which prevents global H3K27me3 deposition by the Polycomb repressive complex PRC2, providing novel insights into the potential function of SMCHD1 in the repression of DUX4 in the early stages of embryogenesis. The SMCHD1 interactome outlined herein can thus provide further direction into research on the potential function of SMCHD1 at genomic loci where SMCHD1 is known to act, such as D4Z4 repeats, the inactive X chromosome, autosomal gene clusters, imprinted loci and telomeres.

## Introduction

Facioscapulohumeral dystrophy (FSHD, [FSHD1; OMIM 158,900 and FSHD2; 158901] is a progressive, often dominantly inherited, muscle disease characterized by facial and upper extremity muscle weakness^1^. It shows marked clinical variability in disease onset and progression. FSHD is caused by incomplete repression of the *DUX4* retrogene in skeletal muscle from the D4Z4 macrosatellite repeat located in the subtelomere of chromosome 4q^2^. The transcription factor DUX4 and its mouse ortholog Dux are involved in zygotic genome activation (ZGA) in cleavage stage embryos while subsequently epigenetically silenced in most somatic tissues^3,4^. In humans, the 3.3 kb large D4Z4 units each containing a copy of the DUX4 open reading frame form tandem repeats of 8–100 copies in non-affected individuals^5^. A highly homologous D4Z4 repeat on chromosome 10q26 exists, but this repeat is generally not associated with FSHD because of the presence of a single nucleotide polymorphism (SNP) disrupting the *DUX4* somatic polyadenylation signal on this chromosome^6–8^. D4Z4 repeats are normally in a repressed chromatin state in somatic cells prohibiting the expression of DUX4 in muscle cells, but become partially derepressed when the repeat is contracted to a size of 1–10 D4Z4 units (FSHD1: ~ 95% of FSHD individuals) and/or in the presence of damaging variants in specific D4Z4 chromatin repressors in combination with an intermediately-sized D4Z4 repeat (FSHD2: ~ 5% of individuals)^2,9–11^. The chromatin modifier which is most frequently found to be mutated in FSHD2 individuals is *Structural Maintenance of Chromosomes Hinge Domain Containing 1* (*SMCHD1*)^12^. SMCHD1 is a component of D4Z4 chromatin^13^, where it contributes to suppression of somatic transcription of *DUX4* through mechanisms largely unknown. While DNA hypomethylation of the contracted D4Z4 repeat is an epigenetic hallmark for repeat contraction-dependent FSHD1, pan-D4Z4 repeat hypomethylation is observed in carriers of a heterozygous pathogenic variant in SMCHD1. While there is a good correlation between SMCHD1 dysfunction and DNA hypomethylation at distinct sites such as the D4Z4 repeat^12^, DNA methylation is not rescued upon restoration of endogenous SMCHD1 levels in FSHD muscle cell cultures^14^. This suggests that SMCHD1 is involved in the establishment or early maintenance of DNA methylation, as was demonstrated in induced pluripotent stem cell (iPSC) culture studies^15^. Despite this failure to recover DNA methylation at D4Z4, restoration of SMCHD1 levels in FSHD muscle cell cultures represses *DUX4* expression in a DNA methylation-independent manner^13,14^, suggesting that DNA methylation-independent chromatin repressor complexes are recruited to D4Z4 through SMCHD1. Additionally, SMCHD1 is important for X chromosome inactivation (Xi) in female mammalian cells, where it assists in higher order chromatin compaction, as well as for discrete autosomal loci other than D4Z4^16–19^. Also on the Xi, genetic variation in SMCHD1 is associated with female-specific changes in X-chromosome methylation^20^. Apart from FSHD2, pathogenic missense variants in SMCHD1 can also cause Bosma Arhinia Microphthalmia Syndrome (BAMS, [OMIM: 603457])^21,22^, a rare severe developmental disorder, but only when these variants occur in the extended ATPase domain of SMCHD1^12^.

Locus specific proteomics studies uncovered the Nucleosome Remodelling and Deacetylase (NuRD) and Chromatin Assembly Factor 1 (CAF1) complexes to be present in D4Z4 chromatin of myocytes^23^. Other studies also identified a number of proteins regulating the D4Z4 chromatin structure^23–25^, though it is generally not known how these complexes are recruited to D4Z4, how they cooperate, and their potential interplay with SMCHD1 has not been studied. To investigate how SMCHD1 is involved in D4Z4 chromatin remodelling, we aimed to identify protein binding partners of SMCHD1 and to study the role of identified complexes in the D4Z4 chromatin structure. As SMCHD1 is ubiquitously expressed, we employed Stable Isotope Labelling of Amino acids in Cell culture Mass Spectrometry (SILAC-MS) of GFP-tagged SMCHD1 in U2OS cells as this provides a robust strategy to identify proteins that potentially interact^26^. We identified novel SMCHD1 interacting partners and validated a subset of them: RuvB Like AAA ATPase 1 (RUVBL1), the cohesin complex component RAD21, and the recently characterized EZH Inhibitory Protein (EZHIP, CXorf67). Furthermore, we provide evidence that this catalogue of probable SMCHD1 interacting proteins can provide guidance into the function of SMCHD1 at D4Z4, and elsewhere in the genome.

## Methods

### Cell culture

U2OS (ATCC, HTB-96), HEK293T (ATCC, CRL-11268) and HeLa (EMBL, Heidelberg, Germany) cells were maintained in Dulbecco’s Modified Eagle Medium (DMEM)-Glutamax, high glucose (Gibco 31966-047) supplemented with 10% Heat-Inactivated Fetal Bovine Serum (HI-FBS) and 1% Penicillin–Streptomycin (PenStrep, Gibco 15140-122). hTERT-RPE-1 cells were a kind gift by H. van Attikum and were maintained in DMEM/F-12-GlutaMAX (Gibco 31331-093) supplemented with 10% HI-FBS and 1% PenStrep. Immortalized FSHD1 (MB073) and FSHD2 (MB200) derived myoblasts (Originally from the Fields Center for FSHD and Neuromuscular Research at the University of Rochester Medical Center) have been described before^23^ and were maintained in Ham’s F10 Nutrient Mix-Glutamax (Gibco 41550-088) supplemented with 20% HI-FBS, 1% PenStrep, 10 ng/ml rhFGF-2 (Promokine C-60240) and 1 mM dexamethasone (Sigma-Aldrich D2915). For differentiation into myotubes, medium of confluent myoblast cultures was replaced with DMEM-Glutamax, high glucose, supplemented with 1% PenStrep and 2% KnockOut serum replacement (Gibco 10828-028) for 24 to 72 h, depending on experimental conditions. All cells used were routinely tested for mycoplasma contamination (Mycoalert, Lonza LT07-318) and cultured in humidified incubators at 37 °C and 5% CO_2_.

Stable GFP-SMCHD1 expressing U2OS cell lines were generated by transient transfection of lentiviral packaging vector pRRL-CMV-GFP-SMCHD1-IRES-Puro (pLV-GFP-SMCHD1) using Lipofectamine 2000 (ThermoFisher 11668019). After selection with 0.3 µg/ml puromycin, cells were seeded as single cells using a BD Aria III cell sorter (BD-Bioscience), gating for a low GFP-positive population. After sufficient cell growth, candidate clones were analysed by western blot, RT-qPCR and fluorescence microscopy to select candidates with moderate expression levels of full length GFP-SMCHD1, localized to the cell nucleus. Clones A7 and G9 were used for all subsequent SILAC Mass-Spectrometry experiments.

For SILAC-MS analysis stable GFP-SMCHD1 U2OS clones A7 and G9 were cultured for at least six passages in Heavy or Light SILAC medium to ensure full isotope incorporation in the proteome. A U2OS clonal culture expressing GFP at comparable level was used as a negative control, and was also cultured in either SILAC medium. SILAC medium consisted of DMEM media minus L-Lysine and L-Arginine (89985 Thermo Scientific) supplemented with 20% Dialyzed FBS for SILAC (88440 Thermo Scientific), 1% PenStrep, 1% Glutamax (35050-038 Life Technologies), L-proline (20 mg/100 ml) (ULM-8333) and either: unlabelled (SILAC-Light medium) L-arginine-HCl (R0–10 mg/100 ml) (ULM-8347) and L-lysine-HCl (K0–20 mg/100 ml) (ULM-8766), or Isotope labelled (SILAC-Heavy medium) L –arginine‐HCl (13C6, 99%; 15N4, 99%—Chemical purity 98%) (R10–10 mg/100 ml) (CNLM-539) and L‐lysine‐2HCl (13C6, 99%; 15N2, 99%—Chemical purity 98%) (K8–20 mg/100 ml) (CNLM-291). Unlabelled proline was added to the culture medium to prevent (labelled) arginine to proline conversion. All labelled and unlabelled amino acids were acquired from Cambridge Isotope Laboratories.

### SILAC-MS sample preparation

U2OS (stably expressing GFP-SMCHD1 or GFP) cells grown in SILAC Heavy or Light medium as described above, were harvested from a full T182 culture flask by dissociation of cells using TrypLE dissociation reagent (12604-013 Life Technologies). Cells were counted, and aliquots of 2.5 × 10^6^ cells were transferred to 1.5 ml microcentrifuge tubes on ice for subsequent nuclear isolation. After washing with PBS and pelleting of cells in a microcentrifuge (2600 RPM), pellets were resuspended in 65 µl Fractionation Buffer 1 (HEPES 25 mM, KCl 5 mM, MgCl_2_ 0.5 mM, DL-Dithiothreitol (DTT) 1 mM supplemented with 1 × cOmplete Protease Inhibitor (11836145001 Roche). Subsequently, 65 µl Fractionation Buffer 2 was added (HEPES 25 mM, KCl 5 mM, MgCl_2_ 0.5 mM, DL-Dithiothreitol (DTT) 1 mM and 2% Igepal CA-630 supplemented with 1 × cOmplete Protease Inhibitor, to reach a final Igepal CA-630 concentration of 1%. Tubes were incubated while tumbling at 4 °C for 15 min. Nuclei were pelleted at 2500 RPM and supernatant was removed, with a part of the supernatant transferred to a clean microcentrifuge tube to serve as a cytoplasmic fraction. Nuclei were washed twice in Fractionation Buffer 3 (1:1 mixture of Fractionation Buffer 1 and Fractionation Buffer 2) and were pooled in a new Eppendorf Protein Lobind tube (EP0030108116, Eppendorf) by resuspension in 300 µl NP40 lysis buffer (50 mM Tris–HCl pH 8.0, 150 mM NaCl, 1% NP-40 (Igepal CA-630) and 1 mM MgCl2, supplemented with 1 × cOmplete protease inhibitors and 20 mM NaF). Lysed nuclei were sonicated in a Bioruptor Pico (Diagenode) for 6 cycles (10 s on, 30 s off) and afterwards supplemented with 500U Benzonase (E1014-25KU EMD Millipore) and an additional 700 µl NP40 lysis buffer. Lysis was completed by incubation at 4 °C while tumbling for 60 min, after which insoluble material was pelleted by centrifugation at 16.000 RPM for 10 min in a cooled microcentrifuge. Protein concentrations in the lysates were determined using Pierce BCA kit (23225 ThermoFisher), and equal amounts of nuclear lysate were incubated with 20 µl washed GFP-Trap Agarose beads (gta-20 Chromotek) slurry for 1.5 h while tumbling. Beads were washed 1 × in NP-40 lysis buffer, 1 × in NP-40 lysis buffer containing 300 mM NaCl, and 2 × in 50 mM ammonium bicarbonate ((NH_4_)_2_CO_3_). At the last wash-step, beads used for corresponding GFP-SMCHD1 IP (e.g. SILAC Heavy) and GFP control IP (e.g. SILAC Light) were combined in a new tube. Mixed samples were then trypsinized overnight by resuspending beads in 250 µl 50 mM Ammonium Bicarbonate containing 2.5 µg sequencing grade trypsin (V5111 Promega). Trifluoroacetic acid (TFA, 40967-10X1ML-F, Honeywell) was added to a final concentration of 1%, and samples were centrifuged for 5 min to pellet insoluble debris. The supernatant was loaded on a Sep-Pak Vac 1 cc (100 mg) tC18 cartridge (WAT036820, Waters), washed twice with 0.1% acetic acid, and eluted using 1 ml 0.1% acetic acid, 60% acetonitrile. Eluates were lyophilized using a RVC 2–25 CD centrifugal vacuum concentrator (Christ) and stored at -80 °C. Per experiment, two U2OS GFP-SMCHD1 clones were used in both Heavy and label swapped Light SILAC medium for an N = 4. The experiment was performed independently two times.

### Mass spectrometry data acquisition

Mass spectrometry data was acquired as in^27^. In brief, Liquid Chromatography was performed on an EASY-nLC 1000 system (Proxeon, Odense, Denmark) connected to a Q-Exactive Orbitrap (Thermo Fisher Scientific, Germany) through a nano-electrospray ion source. The Q-Exactive was coupled to a 15 cm analytical column with an inner-diameter of 75 μm, in-house packed with 1.9 μm C18-AQ beads (Reprospher-DE, Pur, Dr. Maish, Ammerbuch-Entringen, Germany).

The chromatography gradient length was 95 min from 2 to 30% acetonitrile followed by 10 min to 95% acetonitrile in 0.1% formic acid at a flow rate of 200 nL/min. The mass spectrometer was operated in data-dependent acquisition (DDA) mode with a top-10 method. Full-scan MS spectra were acquired in a range from 400 to 2000 m/z at a target value of 3 × 10^6^ and a resolution of 70,000, and the Higher-Collisional Dissociation (HCD) tandem mass spectra (MS/MS) were recorded at a target value of 1 × 10^5^ and with a resolution of 17,500 with a normalized collision energy (NCE) of 25%. The maximum MS1 and MS2 injection times were 20 ms and 60 ms, respectively. The precursor ion masses of scanned ions were dynamically excluded (DE) from MS/MS analysis for 60 s. Ions with charge 1, and greater than 6 were excluded from triggering MS2 analysis.

### Mass spectrometry data analysis

To determine SILAC ratios, LC–MS/MS Raw files were analyzed using MaxQuant software (v1.6.14) according to^28^ using default settings with the following modifications. Two labelings were included, K0R0 and K8R10, for light and heavy medium, respectively. Maximum number of mis-cleavages by trypsin/p was set to 3 and Carbamidomethyl(C) was deactivated as fixed modification. We performed the search against an in silico digested UniProt reference proteome for Homo sapiens (16th Oct 2020). Minimum amount of peptides for quantification was set to 1. Match-between-runs feature was enabled with 0.7 min match time window and 20 min alignment time window.

MaxQuant proteingroups.txt file output was further processed in Microsoft Excel 365 for comprehensive visualization.

Hits with a Heavy over Light ratio of > 1.5 were considered as candidate interactors. Nuclear localization was determined from data available at the human protein atlas^29^. For label swapped experiments (i.e. GFP-SMCHD1 in SILAC light media mixed 1:1 with GFP in SILAC heavy media), Light over Heavy ratios were inverted to aid in correlations with Heavy over Light sets.

### Mass spectrometry data availability

The mass spectrometry proteomics data have been deposited to the ProteomeXchange Consortium via the PRIDE partner repository^30^ with the dataset identifier PXD023993.

### Plasmids and cloning

The full-length open reading frame (ORF) of EZHIP (CXorf67) was amplified from SuSa cell poly-dT cDNA, with primers listed in Supplementary Table s1 using Phusion DNA polymerase (M0530L NEB). Primers included a 5′ SacII and a 3′ BsrGI restriction site, which were used to ligate the product into a pLV-CMV plasmid backbone, which contained either an N-terminal HA- or GFP-tag (pLV-HA-CXorf67 and pLV-GFP-CXorf67, respectively). The constructs were analysed by Sanger sequencing and alignment to reference sequence NM_203407.2. Plasmids encoding short hairpin RNAs (shRNA) were acquired from the Mission shRNA library (TRC1, TRC1.5 and TRC2) (Sigma Aldrich) and are listed in Supplementary Table S4.

### Lentivirus preparation

Lentiviral inoculates were produced using a third-generation lentiviral system utilizing VSV-G as described previously^31^. In brief, HEK293T cells were transfected with 3 helper plasmids (Gag/Pol, Rev and VSV-G) and the transfer plasmid, containing either ORF clones (pLV-CMV plasmids) or shRNAs. Medium was harvested from the HEK293T cells 48 and 72 h post transfection, centrifuged at 2500 RCF for 10 min and filtered using 45 µm Acrodisc Tuffryn syringe filters (4184, Pall corporation). To equalize viral loads upon transduction, viral concentrations in filtered inoculates were measured using the HIV Type 1 p24 ELISA kit (0801111 Zeptometrix). For transduction of myoblasts, DEAE dextran was added prior to addition of the viral inoculates, which were standardized to 3 ng viral particles per cm^2^ of culture surface.

### Co-immunoprecipitation

For co-immunoprecipitation (Co-IP) of overexpressed constructs, HEK293T, U2OS or HeLa cells were transfected using polyethylenimine (PEI) (23966-2 Polysciences) with constructs as indicated. 48 h post transfection, cells were washed with cold PBS and lysed in NP40 lysis buffer (50 mM Tris–HCl pH 8.0, 150 mM NaCl, 1% Igepal CA-630) supplemented with cOmplete Protease Inhibitors and NaF. Lysates were incubated while tumbling at 4 °C for 15 min, and were subsequently centrifuged at max speed in a cooled microcentrifuge to pellet insoluble debris. Initially, samples were incubated with 200U Benzonase for 1 h before sedimentation of debris. As this did not affect the co-precipitation of any of the selected interacting proteins (data not shown), this step was omitted in follow-up experiments. An input sample was acquired, which was supplemented with an appropriate volume of 5 × Laemmli Sample Buffer (10% SDS, 50% glycerol, 10% β-mercaptoethanol and 0.05% bromophenol blue in 300 mM Tris pH 6.8). The remainder of the lysate was incubated with either 8 µl washed HA-bead slurry (A2095-1ML, Sigma-Aldrich), 8 µl washed Flag M2 Affinity gel (A2220, Sigma-Aldrich), or 10 µl washed GFP-Trap Agarose beads (gta-20 Chromotek) for 2 h while tumbling at 4 °C. Beads were washed 3 × 5 min with lysis buffer, and subsequently boiled in 40 µl 2 × Laemmli Sample buffer before western blot analysis. For endogenous Co-IP experiments, lysis and processing steps were nearly identical, except for the use of a mixture of 20 µl Dynabeads protein A and protein G (3:1 ratio) (10002D and 10003D, Thermo Fisher) per sample. Antibodies (1 µg per Co-IP sample) were conjugated to washed Dynabeads for 1 h prior to addition of cell lysates as indicated.

### PAGE and Western blot analysis

For polyacrylamide gel electrophoreses (PAGE) and western blot analysis of total cell lysates, cells were washed with PBS and lysed in 1 × Laemmli Sample Buffer (2% SDS, 10% glycerol, 60 mM Tris pH6.8) and boiled at 95 °C. Samples were equalized after determination of protein content by Pierce BCA kit and supplemented with 2% β-mercaptoethanol and 0.01% bromophenol blue. Directly prior to loading of samples on protein gels, samples were boiled again at 95 °C. Samples were separated using Criterion TGX gradient gels (5671094 and 5671093 Bio-Rad) and subsequently transferred to Immobilon-FL PVDF membranes (IPFL00010, EMB Millipore). Membranes were blocked in 4% skim milk (70166-500G Sigma Aldrich) in PBS, and probed with primary antibodies in Takara Immunobooster 1 (T7111A Takara). After washing, membranes were incubated with secondary antibodies donkey-α-Rabbit IRDye-800CW (926–32,213), donkey-α-Mouse IRDye-680RD (926–68,072), Westernsure HRP goat-α-Mouse (926–80,010) or Westernsure HRP goat-α-Rabbit (926–80,011) (All from LI-COR). Immunocomplexes containing IRdye were scanned on an Odyssey Classic infrared imaging system (LI-COR) for visualization and subsequent analysis using the manufacturers application software (V3.0). HRP probed membranes were incubated in Pierce ECL Plus Western Blotting Substrate (32132 ThermoFisher) for 5 min and imaged using an Amersham Imager 680 (GE Healthcare Life sciences).

### RT-qPCR analysis

For quantitative real-time PCR analysis, cells were lysed directly in QIAzol Lysis reagent (79306 Qiagen) (350 µl per 6 well), and total RNA was extracted using the Direct-zol RNA miniprep kit (R2062 Zymo Research), as per manufacturer’s instructions, including DNAseI treatment. RNA was analysed using a nanodrop ND-1000 (Themofisher) to assess RNA concentration, as well as A_260_/A_230_ and A_260_/A_280_ ratios. Up to 2 µg total RNA was used in a 20 µl reaction for first strand cDNA synthesis using the RevertAid First Strand cDNA Synthesis Kit (K1622 ThermoFisher), using oligo dT primers as per manufacturer’s instructions. Generated cDNA was treated with 2 units Ambion RNase H (AM2293 ThermoFisher) for 20 min at 37 °C and diluted to a final volume of 100 µl by addition of RNase-free MQ. Gene expression was measured by using gene specific primers (IDT) (See Supplementary Table S3) and iQ SYBR-Green Supermix (1708887 Bio-Rad) run in a CFX-384 Real-Time PCR system (Bio-Rad) manually pipetted into 384 wells plates (Framestar 480/384, 4ti-0381 4titude). Reaction volume was 10 µl (5 µl 2 × SYBR green Supermix, 1 µl forward primer 10 µM, 1 µl reverse primer 10 µM, 2 µl cDNA and 1 µl RNase-free MQ) with each cDNA/primer combination measured in a technical triplicate. Cycling conditions were as follows: 1: 95 °C 5:00; 2: 95 °C 0:10; 3: 60 °C 0:30 (plate read); 4: Go to step 2 39 additional times; 5: melt curve 60 °C to 95 °C, 0.5 °C increase per cycle of 0:05. Data were analysed using the accompanying CFX-manager V3.1 software, which calculated Cq values and baseline in ‘auto calculation’ mode and normalized to housekeeping genes (HKG) using the ΔΔCt method, followed by statistical analysis in GraphPad Prism Version 8. Specificity of reactions was assessed by examining melt curves peak and position acquired in step 5 of the RT-qPCR protocol as described. Internal QC functions of CFX-manager were used to evaluate reliability of the assay. As previously described^11,14,32,33^, HKG *GUSB* was used for normalization purposes in Supplementary Figures 1B, 4E and 5. HKG genes *GUSB* and *GAPDH* were used for normalization purposes in Fig. 3B and Supplementary Figure 6A.

### Immunofluorescent (IF) microscopy

For immunofluorescent (IF) microscopy, myoblasts were seeded on glass #1.5 coverslips (Hecht Assistant), coated with collagen I. Cells were fixed with 4% Paraformaldehyde (15710 Electron Microscopy Sciences) for 10 min, permeabilized with 1% Triton X-100 for 15 min, and blocked with 0.1% Bovine Serum Albumin (BSA) in PBS containing 0.05% Tween-20 (PBS-T). Slides were incubated for 1 h in primary antibody (Rabbit-α-DUX4, E14-3, Geng et al. 2011^34^) (Mouse-α-myosin, University of Iowa hybridoma bank MF20, Bader et al. 1982^35^) dissolved in blocking buffer, and subsequently washed 3 times 5 min with PBS-T. Slides were incubated with secondary antibodies Donkey-α-Rabbit-Alexa488 (A21206, Molecular probes (Invitrogen) 1:500), Donkey-α-Mouse-Alexa594 (A21203, Molecular probes (Invitrogen) 1:500) and DAPI (62248 ThermoFisher, 1:1000) in blocking buffer for 1 h, washed 3 times for 5 min with PBS-T and air-dried. Coverslips were mounted on microscopy slides in Prolong Gold (P36930 ThermoFisher). Imaging was performed using a Leica SP8 upright confocal system, or a Leica SP8 White Light Laser (WLL) inverted confocal system (Leica Microsystems) using a 100 × or 40 × objective, respectively. Quantification of microscopy data was performed on automated tile-scans (9 × 9 image grid for a total of 81 images per acquisition) using the SP8 WLL, of which the starting location was randomly picked on the coverslip. Acquired data were analysed for percentage of DUX4 positive cells and myogenic fusion index using a Cell Profiler (4.0.6) analysis pipeline followed by data processing in R-Studio (1.2.5042). Outlines of nuclei were manually curated if necessary. Positive counts of DUX4 nuclei, and fusion index, per image are used for the plots in Fig. 3 and Supplementary Figure 6.

### Chromatin immunoprecipitation (ChIP)

Chromatin immunoprecipitation (ChIP) was performed as described previously^9,13^. In brief, cells were crosslinked for 10 min by addition of formaldehyde to culture media, which was quenched with addition of Glycine to a final concentration of 125 mM. Cells were collected and lysed in NP-ChIP-buffer (150 mM NaCl, 5 mm EDTA, 0.5% Igepal CA-630, 1% Triton X-100 in 50 mM Tris–HCl pH 7.5) to isolate cell nuclei, which were sheared using a Bioruptor Pico (Diagenode) (Myotubes: 25 cycles, 30 s on/off in 1.5 ml Bioruptor Pico sonication tubes (Diagenode)). 30 µg sheared precleared chromatin was used per ChIP for chromatin binding proteins. Antibodies were incubated with chromatin overnight, antibodies used are listed in Supplementary Table S2. Chromatin-antibody complexes were captured by addition of Protein A/G Sepharose beads (17-5280-01, 17-0618-01—GE Healthcare (3:1 ratio A/G beads)) for 2 h. Beads were washed with low salt buffer (0.1% SDS, 1% Triton-X100, 2 mM EDTA, 150 mM NaCl in 20 mM Tris–HCl pH 8), high salt buffer (0.1% SDS, 1% Triton-X100, 2 mM EDTA, 300 mM NaCl in 20 mM Tris–HCl pH 8), lithium chloride wash buffer (0.25 M LiCl, 1% Igepal CA-630, 1% Sodium deoxycholate, 1 mM EDTA in 10 mM Tris–HCl pH 8) and twice with TE-wash buffer (1 mM EDTA in 10 mM Tris–HCl pH8 ). ChIP DNA was eluted from the beads by addition of 10% Chelex-100 (142–1253 Bio-Rad) and heating to 95 °C for 10 min. Input samples corresponding to 10% of input material were isolated using Phenol–Chloroform extraction. Samples were analysed by RT-qPCR using primer sets specific for genomic regions of interest (Supplementary Table S3) using iQ SYBR-Green Supermix (Bio-Rad) run in a CFX-384 Real-Time PCR system (Bio-Rad). ChIP samples were normalized to input by use of ΔCt calculations. Statistical analysis was performed in GraphPad Prism Version 8.

### Co-expression analysis

Publicly available datasets were downloaded and analysed for expression of genes of interest as outlined. Datasets from early mouse embryogenesis generated by Wu et al. were downloaded from the GEO database: GSE66582^36^. Datasets from cultured control and FSHD myocytes generated by Yao et al. were downloaded from the GEO database: GSE56787^37^. Datasets for myogenic differentiation generated by Trapnell et al. were downloaded from the GEO database: GSE52529^38^.

## Results

### A proteomics approach identifies novel interaction partners of SMCHD1

**Figure 1:**
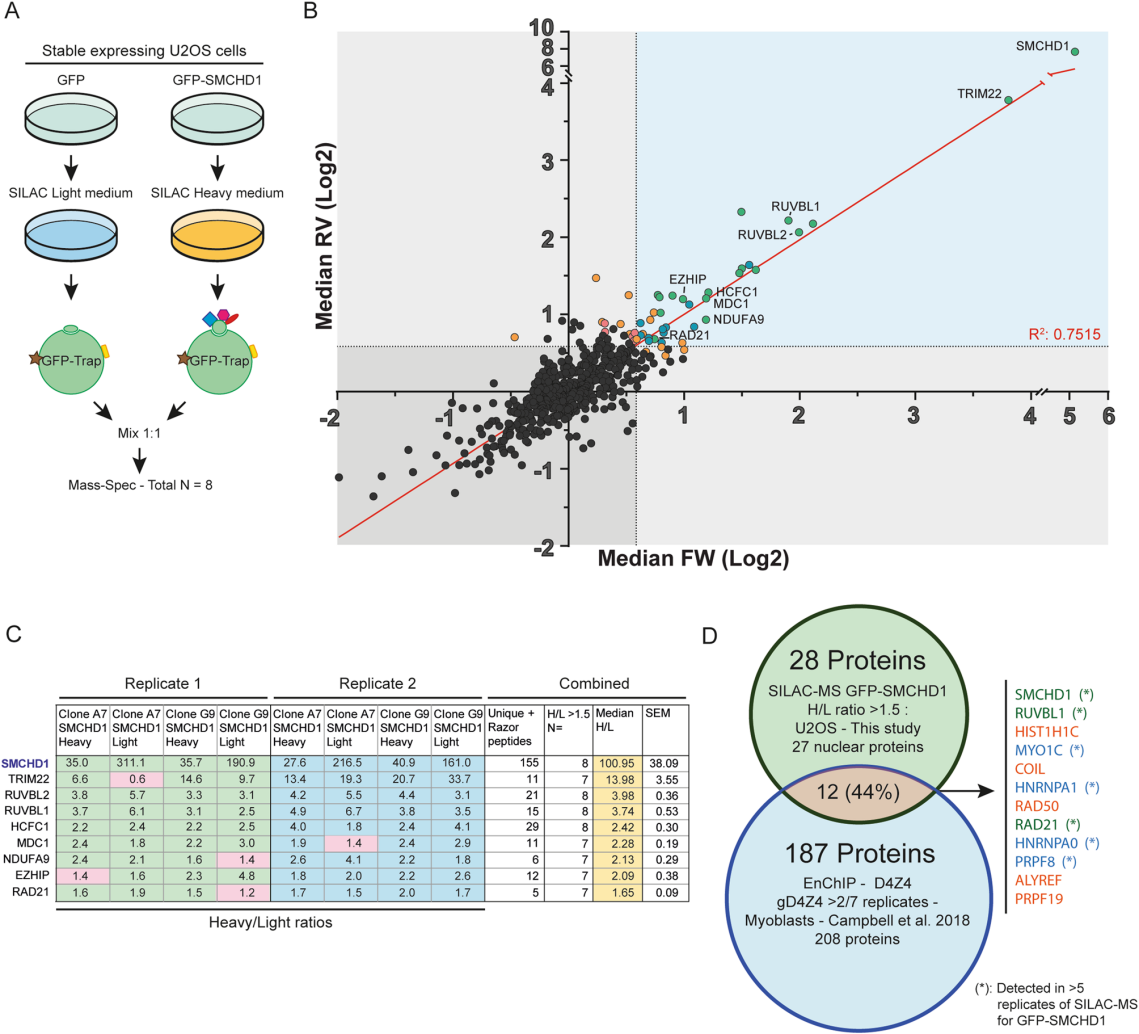
SILAC-MS identifies novel binding partners of SMCHD1. (**A**): Experimental set-up of the SILAC-MS screen in U2OS cells stably expressing GFP or GFP-SMCHD1. (**B**): Visual representation of potential SMCHD1 protein interaction partners. Median Log2 transformed Heavy GFP-SMCHD1 SILAC ratios compared to the light GFP-only controls (FW) of 4 replicate sets are plotted on the X-axis, against their corresponding Light GFP-SMCHD1 SILAC ratios compared to the heavy GFP-only controls (RV). Colour coding denotes the number of samples out of 8 replicates in which a protein’s H/L was > 1.5. Green: 8–7/8; Blue: 6/8; Orange: 5/8; Red: 4/8; grey: < 4/8. The identity of nuclear proteins with a median H/L ratio > 1.5 identified in 7 or 8 replicates are indicated. The dotted lines indicate the H/L ratio threshold of 1.5 (Log2 transformed: 0.585). The correlation of the median FW and RV (R-square) was calculated to be 0.7515 (red line). (**C**): Table representation of the 9 proteins identified in the SILAC-MS analysis with highest confidence. Individual H/L ratios of each replicate samples and peptide counts are listed, as well as the median H/L ratio used in (**B**). (**D**): Venn diagram showing overlap between SMCHD1 interaction partners identified in this study (all nuclear proteins with median H/L ratio > 1.5), compared to D4Z4 chromatin components identified by Campbell et al. The 12 overlapping proteins identified are listed on the right and coloured as outlined in (**B**). Asterisk indicates detection in > 5 replicates of GFP-SMCHD1 SILAC-MS.

To identify novel binding partners of SMCHD1 we employed SILAC-MS as this technique enables the acquisition of highly specific semi-quantitative interaction spectra (Fig. 1A)^39^. We generated two independent clonal U2OS cell lines expressing GFP-SMCHD1 at low levels in the nucleus (Supplementary Figures 1A-C). After GFP affinity purification of nuclear protein fractions (Supplementary Figures 1D and 1E), trypsin digested peptides were analysed by mass spectrometry. An U2OS line expressing comparable levels of GFP was used for the control (GFP-SMCHD1-negative) dataset. Each purification was performed twice, including a SILAC heavy/light media label swap. Thus, we obtained mass spectra of putative SMCHD1 protein interactors from 8 GFP-SMCHD1 cultures, matched with corresponding GFP only controls (Fig. 1A). Heavy/Light (H/L) ratios were calculated for all identified proteins after which the median log2 transformed ratios of the 4 heavy labelled GFP-SMCHD1 samples (Forward) and the 4 light labelled GFP-SMCHD1 (Reverse) replicates were plotted against each other (Fig. 1B). The calculated R-square value when comparing median values of the identified proteins in the forward and reverse samples was 0.7515, indicating that the label-swap did not adversely affect identified interacting proteins. Nuclear proteins^29^ with a median H/L ratio of > 1.5 were considered as potential interactors of SMCHD1 (Fig. 1B,C). We further defined strong candidate interaction partners as proteins which were consistently assigned a H/L ratio of > 1.5 in at least 7 out of 8 replicates (indicated in green in Fig. 1B, listed in Fig. 1C). Lower confidence interactors are indicated in blue: (6 out of 8 replicates), orange: (5 out of 8 replicates) and red: (4 out of 8 replicates). Proteins with H/L ratios of > 1.5 in 3 or less replicates are coloured grey. Based on these criteria, we report 28 potential nuclear interacting partners of SMCHD1, with SMCHD1 itself and 8 other proteins robustly represented in our dataset (Fig. 1C) (Supplementary Table S5). Individual plotting of each replicate clonal line label-swapped forward and reverse dataset shows that most high confidence interactors are robustly identified in the majority of replicates (Supplementary Figure 2).

SMCHD1 binds D4Z4 repeats, where it represses DUX4 in somatic cells^13^*.* To gain insight in the complexes interacting with, and required for, SMCHD1 function at the D4Z4 repeat in somatic cells, we compared our list of putative SMCHD1 interaction partners with proteins previously identified to bind D4Z4 in myoblasts by enChIP analysis (Fig. 1D)^40^. We observed 12 proteins present in both datasets, some of which are known chromatin-associated proteins such as RUVBL1 and RAD21 (Fig. 1D). The presence of these proteins at D4Z4 (enChIP dataset), while also being potential interactors of SMCHD1 (this study) suggests a collaborative role in the maintenance of D4Z4 repression.

### Validation of high confidence SMCHD1 interacting proteins

**Figure 2:**
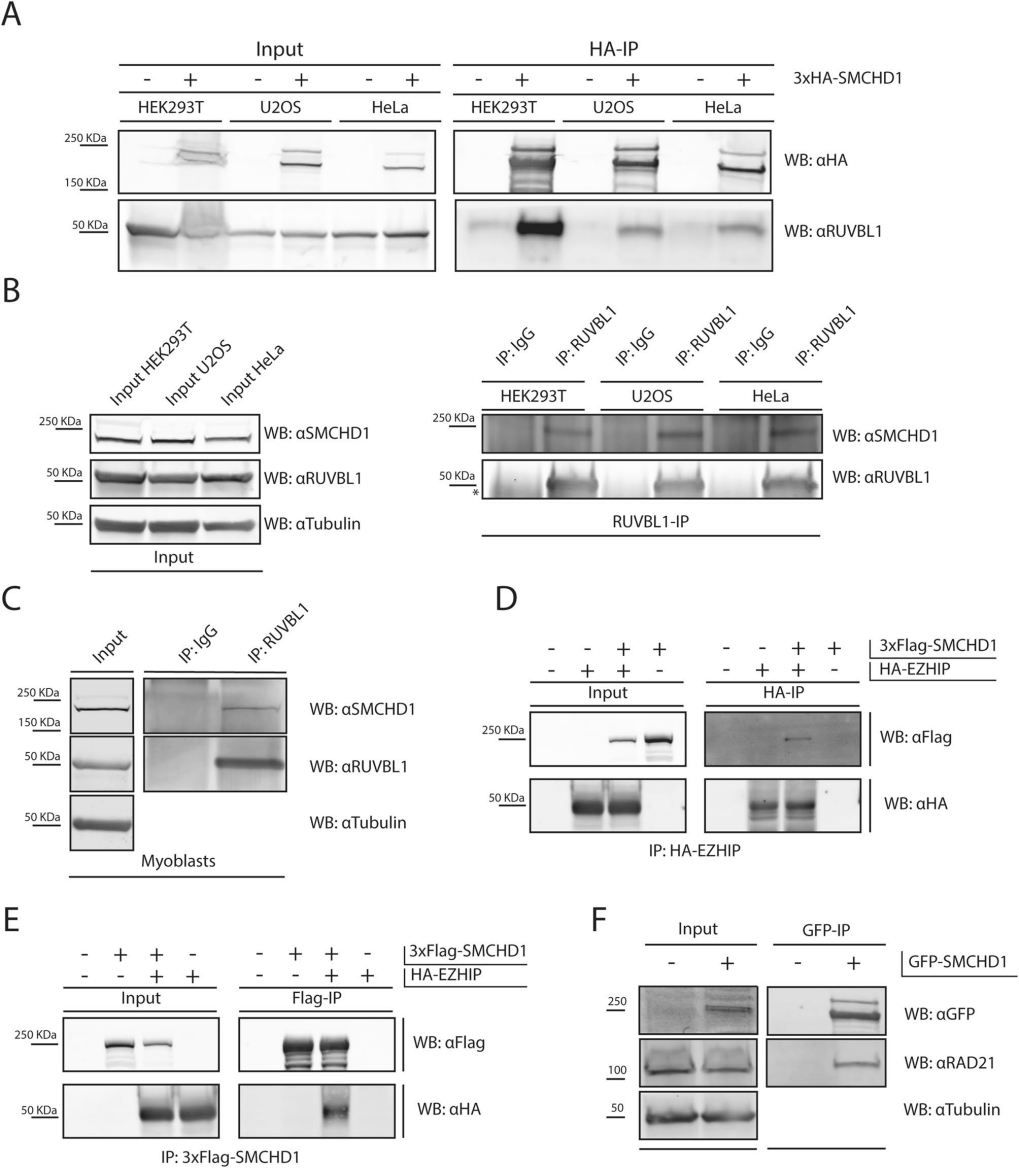
Validation Co-IPs confirm the interaction of SMCHD1 with RUVBL1 and EZHIP. (**A**): Western blot after HA-IP in various cell types. 3xHA-SMCHD1 was ectopically expressed in HEK293T, U2OS and HeLa cells as indicated. Cells were lysed, and HA-tagged proteins were precipitated using HA-agarose beads. Probing the membrane for endogenous RUVBL1 shows Co-IP with SMCHD1 in all cells analysed. (**B**): Western blot after immunoprecipitation of endogenous RUVBL1 in HEK293T, U2OS and HeLa cells. Upon purification of RUVBL1, endogenous SMCHD1 can be detected in the IP fraction for each cell type. The asterisk denotes the presence of IgG heavy chains present in the sample. (**C**): Western blot after immunoprecipitation of endogenous RUVBL1 in control myoblasts. Upon purification of RUVBL1, endogenous SMCHD1 can be detected in the IP fraction. (**D**): HA-IP in HEK293T cells after ectopic expression of 3xFLAG-SMCHD1 and HA-EZHIP. Immunoreactivity for FLAG-tagged SMCHD1 is only detected in the IP fraction in the presence of HA-EZHIP. (**E**): FLAG-IP in HEK293T cells after ectopic expression of 3xFLAG-SMCHD1 and HA-EZHIP. Immunoreactivity for HA-tagged EZHIP is only detected in the IP fraction in the presence of 3xFLAG-SMCHD1. (**F**): Western blot analysis after GFP-IP in HEK293T cells shows co-immunoprecipitation of RAD21 with GFP-SMCHD1.

RUVBL1 has been described to partake in various multimeric complexes, functioning as transcriptional and/or chromatin modifier, either with or without its homolog RUVBL2. Among these complexes are INO80, NuA4, SWR1, TIP60-P400, PAQosome (R2TP) and BAHD1^41–43^. No other components of these complexes have been described to bind to D4Z4, nor were they identified as SMCHD1 interactors in our SILAC-MS approach. To confirm protein interaction between SMCHD1 and RUVBL1, we first validated the putative interaction by GFP-pulldown for GFP-SMCHD1 in our stable GFP-SMCHD1 U2OS cells. Western blot analysis detected endogenous RUVBL1 only in the presence of GFP-SMCHD1 (Supplementary Figure 3A). To assess whether this interaction is U2OS specific, we employed co-immunoprecipitation (Co-IP) assays in 3 different cell lines. 3xHA-SMCHD1 was ectopically expressed in HEK293T, U2OS and HeLa cells, after which 3xHA-SMCHD1 and interacting proteins were purified from cell lysates using HA-agarose beads. In all cell lines, RUVBL1 was co-immunoprecipitated with 3xHA-SMCHD1 confirming a robust interaction between both proteins (Fig. 2A and Supplementary Figure 3B). Similar results were obtained by immunoprecipitating ectopically expressed GFP-SMCHD1 (Supplementary Figure 3B). We immunoprecipitated endogenous RUVBL1 from HEK293T, U2OS and HeLa cells by using a RUVBL1-specific antibody, and were able to detect endogenous SMCHD1 by western blot (Fig. 2B). Finally, we performed endogenous RUVBL1 immunoprecipitation in myocytes, and were able to detect the co-precipitation of endogenous SMCHD1 by western blot (Fig. 2C). Together, these data confirm that SMCHD1 interacts with RUVBL1 in various cell types. Similar to RUVBL1, RUVBL2 co-immunoprecipitated with ectopically expressed GFP-SMCHD1 in both HEK293T and U2OS cells (Supplementary Figures 3C,D), suggesting that this interaction can occur in different cellular backgrounds.

To further strengthen our confidence in the specificity of top SMCHD1 interacting proteins identified by MS, we also validated the putative interaction of SMCHD1 with the recently characterized EZH Inhibitory protein (EZHIP (CXorf67)). EZHIP prevents the deposition of H3K27me3, and is mis-regulated in various cancers which are hallmarked by a global lack of H3K27me3^44–47^. We transiently expressed 3xFLAG-SMCHD1 and HA-EZHIP in HEK293T cells, and after purification of HA-EZHIP, we were able to detect 3xFLAG-SMCHD1 in the IP fraction (Fig. 2D). Reciprocally, purification of 3xFLAG-SMCHD1 from HEK293T lysates reproducibly co-immunoprecipitated HA-EZHIP (Fig. 2E). Finally, IP of endogenous EZHIP in HEK293T, U2OS and HeLa cells resulted in co-precipitation of SMCHD1 (Supplementary Figure 3E). We noted that in U2OS, the observed molecular weight deviates from the expected size of 52 kDa (Supplementary Figure 3E).

RAD21 was found in 3 out of 8 replicates in our MS as a potential interactor of SMCHD1. RAD21 is part of the cohesin complex, together with structural maintenance of chromosomes 1 (SMC1) and 3 (SMC3). This complex localizes to D4Z4^23,48^, but knockdown of RAD21 seems to have little effect on *DUX4* expression in FSHD myocytes^13^. RAD21 co-immunoprecipitates with GFP-SMCHD1 in HEK293T cells (Fig. 2F), again confirming the SILAC-MS results.

Altogether, our ability to co-immunoprecipitate SMCHD1 and proteins of interest identified by SILAC-MS in different cell types supports the robustness of the captured SMCHD1 interactome.

### EZHIP expression correlates with loss of H3K27me3, but EZHIP is not expressed in myocytes

EZHIP binds to PRC2 complex proteins such as EZH2 and SUZ12, and high levels of EZHIP inhibit EZH2 activity resulting in a loss of the repressive histone modification H3K27me3^44–47^. We confirmed the loss of H3K27me3 upon EZHIP overexpression in hTERT-RPE1 cells, a cell line which has been used in the past for studying the relationship between the H3K27me3-marked Xi and SMCHD1^49^, (Supplementary Figure 4A): 77.3% of cells overexpressing EZHIP showed low H3K27me3 immunoreactivity, compared to 23.5% of cells not overexpressing EZHIP (Supplementary Figure 4B). The low levels of H3K27me3 in U2OS (Supplementary Figure 4C) can be explained by the high levels of EZHIP in these cells (Supplementary Figure 4D), as knockdown of EZHIP in U2OS by siRNA leads to an increase of global H3K27me3 as determined by IF (Supplementary Figure 4C), similar to data obtained by Piunti et al.^47^. However, analysis by western blot and RT-qPCR showed that EZHIP is not expressed in myoblasts and myotubes, excluding a role for EZHIP in the regulation of the D4Z4 chromatin structure in skeletal muscle (Supplementary Figures 4D, 4E). While expressed at low levels in SuSa and HEK293T cells (Supplementary Figures 4D), similar to our previous observation (Supplementary Figure 3E), this analysis also showed that in U2OS, EZHIP has a higher molecular weight than the expected protein size of 52 kDa. This higher molecular weight protein could be due to a fusion between MBDT1 and EZHIP, which has been reported to occur in certain types of cancer^50^. However, PCR analysis of U2OS cDNA and genomic DNA showed no conclusive evidence for the occurrence of this specific MBTD1-EZHIP fusion (data not shown). Additional 5’- and 3’-RACE PCR analysis was also unsuccessful in identifying a fusion product (data not shown). Despite the elusive nature of the EZHIP protein in U2OS, the above-described knock down studies corroborate its functionality as an EZH2 inhibitor. Furthermore, stimulated emission depletion (STED) super-resolution microscopy analysis (Supplementary Figure 4F) confirmed the presence of EZHIP in the nucleoplasm, while it is largely excluded from nucleoli (Supplementary Figures 4A, 4F).

Altogether our study confirms that a functional form of EZHIP is highly expressed in U2OS cells and that it interacts with SMCHD1 in HEK293T, HeLa and U2OS cells, but is not present in muscle cells.

### RUVBL1 is involved in DUX4 transcriptional regulation in muscle cells

**Figure 3:**
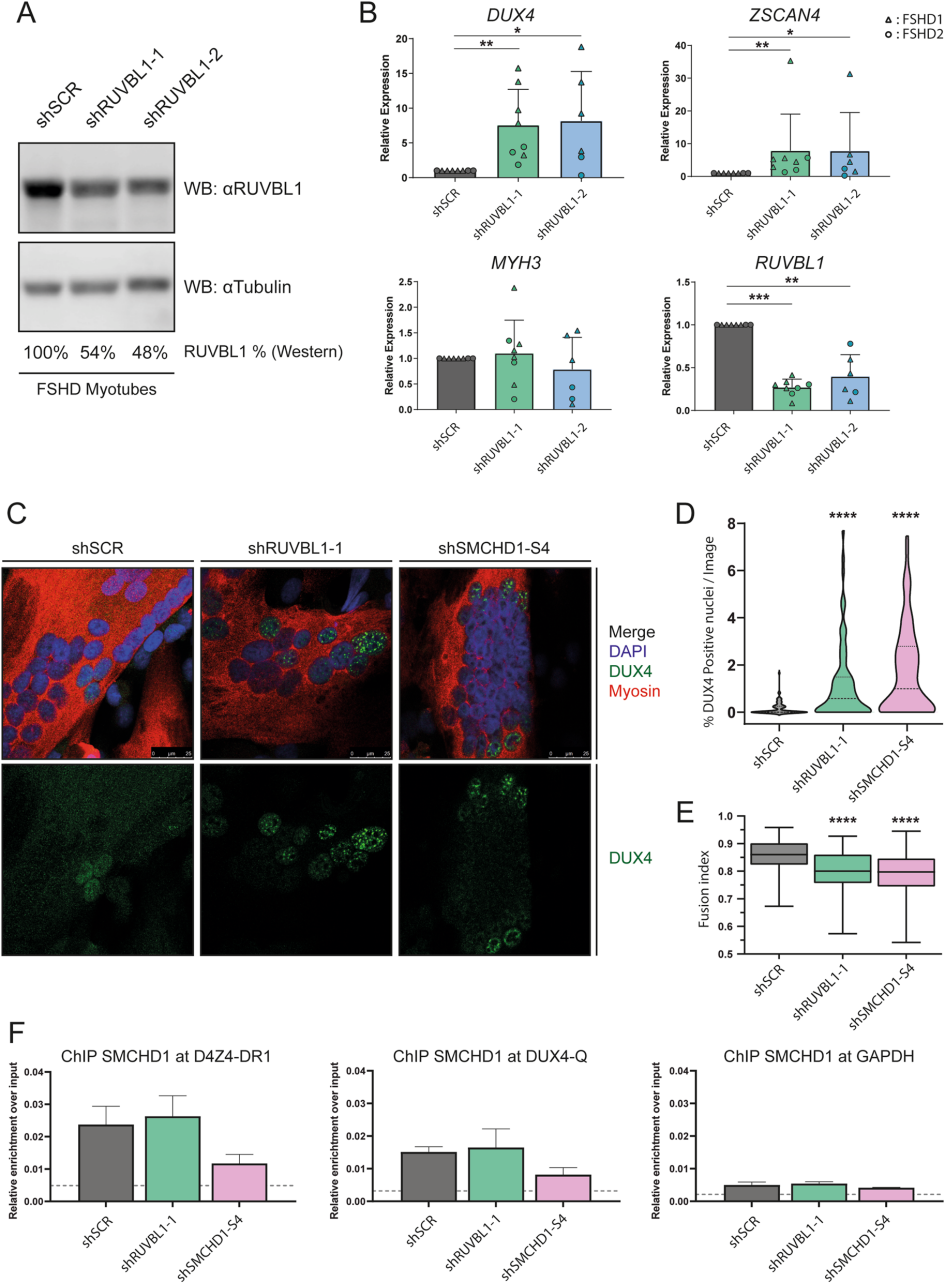
Decreased RUVBL1 levels in FSHD myotubes leads to increased DUX4 expression. (**A**): Representative western blot showing reduced RUVBL1 protein levels in FSHD myotube samples treated with independent shRNAs targeting RUVBL1 (shRUVBL1-1 and shRUVBL1-2) compared to scrambled control shRNA (shSCR). Tubulin was used as loading control. Percentages indicate levels of RUVBL1 protein normalized to tubulin. (**B**): RT-qPCR analysis of FSHD myotube samples corresponding to western blot in (**A**). Expression of *DUX4* and DUX4 target gene *ZSCAN4* is significantly increased upon *RUVBL1* depletion (shRUVBL1-1: N = 8, shRUVBL1-2: N = 6). FSHD1: Triangles, FSHD2: Circles. (**C**): Confocal immunofluorescent microscopy analysis of FSHD derived myotubes depleted for RUVBL1 or SMCHD1. Top panels show nuclei (DAPI—Blue), DUX4 (Green) and Myosin (Red). Bottom panels show DUX4 only. Scalebar: 25 µm. (**D**): Violin plot of high content analysis for DUX4 positive nuclei of slides shown in (**﻿C**). (N = 3, total nuclei counted: shSCR: 55,642; shRUVBL1: 29,721; shSMCHD1-S4: 26,423). (**E**): Box and whisker plot of myogenic fusion index determined in slides from C by high content analysis. (N = 3). (**F**): ChIP-qPCR analysis of SMCHD1 occupancy at two D4Z4 loci and negative control locus *GAPDH*, relative to input. Knockdown of RUVBL1 does not lead to loss of SMCHD1 at D4Z4 in FSHD myotubes. Dotted line indicates enrichment for IgG control. (N = 2). Error bars: SEM. (*P value < 0.05, **P value < 0.01, ***P value < 0.001, ****P value < 0.0001, NS: Not-Significant—Kruskal–Wallis One-Way ANOVA).

We next sought to investigate the role of RUVBL1 in D4Z4 chromatin repression since RUVBL1 was both identified in our SILAC-MS and in the previously published D4Z4 enChIP^40^. Expression analysis of *RUVBL1* (or *RUVBL2* for completeness) showed no significant difference in steady state transcript levels between myoblasts and myotubes from primary control and FSHD1 or FSHD2 muscle cell cultures (Supplementary Figure 5). Knockdown of RUVBL1 using shRNAs in immortalized, DUX4 expressing FSHD1 and FSHD2 myotubes showed that upon efficient knockdown of RUVBL1, as confirmed on western blot (Fig. 3A), *DUX4* mRNA expression levels were significantly increased (Fig. 3B). Transcript levels of *ZSCAN4*, a target gene of DUX4, were also upregulated suggesting that the expressed *DUX4* transcript is translated into a functional protein (Fig. 3B). We also tested the expression levels of three other DUX4 target genes; *KHDC1L, MBD3L2* and *TRIM43* (Supplementary Figure 6A). While *MBD3L2* and *TRIM43* were significantly upregulated in both RUVBL1 shRNA treated samples, *KHDC1L,* was not significantly upregulated upon RUVBL1 knockdown in the shRUVBL1-2 treated samples (Supplementary Figure 6A). Myogenic differentiation marker *MYH3*, as well as *RUVBL2* and *SMCHD1* were not significantly altered upon depletion of *RUVBL1*, while we noted a slight change in *MYOG* expression for one specific *RUVBL1* shRNA (Fig. 3B, Supplementary Figure 6A). We also attempted to knock down *RUVBL2* to study the effects on *DUX4* expression but noticed considerable loss of viability of myoblasts*,* prohibiting this analysis. This is in agreement with studies in which RUVBL2 was identified as an essential gene in human cells^51^.

In FSHD myocyte cultures, DUX4 protein can be detected in 1:1000 up to 1:100 nuclei^52^. To visualize the effect of knockdown of *RUVBL1* and *SMCHD1* on DUX4 expression in individual nuclei, we performed DUX4 immunofluorescent microscopy analysis of three independent immortalized FSHD myotube cultures after shRNA-mediated knockdown of RUVBL1. Depletion of SMCHD1 and RUVBL1 was again confirmed by western blot analysis (Supplementary Figure 6B). We observed a gradient of DUX4 immunoreactivity in the myonuclei of mock and RUVBL1 or SMCHD1 depleted myotubes, supporting earlier observations that DUX4 from a low number of expressing nuclei can spread to neighbouring nuclei (Fig. 3C)^53–55^. Quantification of > 110.000 independent myonuclei (Supplementary Figure 6C) showed a significant 7.6-fold and 11.0-fold increase in the mean number of DUX4 expressing myonuclei after RUVBL1 and SMCHD1 depletion, respectively, compared to myotubes transduced with a scrambled control shRNA (shSCR; Fig. 3D, Supplementary Figure 6D). We observed a mild but significantly reduced mean myogenic fusion index for both RUVBL1 and SMCHD1 depleted cultures compared to shSCR (1.07-fold and 1.09-fold, respectively) (Fig. 3E, Supplementary Figure 6E). As DUX4 levels are known to increase during myogenic differentiation^13^, the increases in DUX4 upon RUVBL1 or SMCHD1 knock down seem to be a direct consequence of their repressive role on the D4Z4 repeat. Finally, we asked whether recruitment of SMCHD1 and RUVBL1 to D4Z4 chromatin is interdependent. Since available RUVBL1 antibodies were not ChIP grade (data not shown), we assessed the recruitment of SMCHD1 to D4Z4 at two different sites after shRNA-mediated knockdown of either RUVBL1 or SMCHD1. We observed no detectable loss of SMCHD1 protein at the D4Z4 repeat upon knockdown of RUVBL1 (Fig. 3F), while knockdown of SMCHD1 itself did lower its enrichment at D4Z4, confirming SMCHD1 antibody specificity.

### Co-regulation of SMCHD1 interactors during early development

DUX4 and its mouse homolog Dux are transcription factors involved in ZGA, and the mechanism of their activation and silencing is only partly understood^3^. As Smchd1 was recently suggested to facilitate the repression of *Dux* after ZGA^56^, and to facilitate de novo methylation of D4Z4 in pluripotent cells^15^ we analysed publicly available RNA-seq data from Wu et al.^36^ describing gene expression levels at various stages of early mouse development and plotted the expression of *Dux, Smchd1* and the proteins identified to interact with SMCHD1 with high confidence (≥ 7 × identified by SILAC-MS) (Supplementary Figure 7). *Ezhip* is expressed at high levels in the oocyte, zygote and early 2-cell stages, and is almost completely lost upon reaching the 4-cell stage (Supplementary Figures 7A, 7B). The stage of repressed *Ezhip* expression from the 2-cell stage is similar to the expression pattern seen for *Dux* and Dux downstream targets such as *Zscan4* (Supplementary Figures 7D, 7 J, 7 K). We noted that the presence of some other nuclear high confidence interactors (Ruvbl1, Ruvbl2, Mdc1 and Hcfc1) increases after the peak of expression of Dux at the 2 cell stage (Supplementary Figure 7).

We extended the analysis of available RNA-seq datasets to data from cultured human control and FSHD derived myoblasts and myotubes (Supplementary Figure 8)^37^, skeletal muscle biopsies from control and FSHD individuals (Supplementary Figure 9)^37^ and control myocytes during differentiation (Supplementary Figure 10)^38^ as DUX4 expression is dynamically regulated during muscle cell differentiation^36^. These datasets confirmed the lack of expression of *EZHIP* in all myogenic samples (Supplementary Figures 8, 9 and 10). No consistent changes in gene expression were observed between control and FSHD for any of the SMCHD1 interacting proteins identified (Supplementary Figures 8 and 9). The expression levels for genes which were present in our RT-qPCR analysis of cultured myocytes (Supplementary Figure 5) is largely in agreement with the RNA-seq data (Supplementary Figure 8 and 9). Upon myogenic differentiation we noted a mixed picture of interactors trending upwards or downwards in expression with several D4Z4-associated proteins (SMCHD1, RUVBL1, HNRPA1, HCFC1 and PRPF8) showing lower expression in terminally differentiated muscle cells in culture (Supplementary Figure 10).

## Discussion

In this study we describe the identification and validation of novel protein binding partners of SMCHD1 in human cells, and investigate the function of these interactions in different cell types. We focused our validation set on proteins that bind to D4Z4 and might have a role in DUX4 regulation, as the epigenetic deregulation of the D4Z4 repeat in somatic cells and inappropriate expression of DUX4 in skeletal muscle is the cause for FSHD. In total, we identified 28 nuclear proteins that potentially interact with SMCHD1. Of these, 12 have previously shown to be present in D4Z4 chromatin. Previous SMCHD1 protein studies suggested a role for SMCHD1 in various processes such as gene silencing, telomere maintenance and chromatin regulation^57–59^. It will be interesting to examine the involvement of the novel interaction partners described here in the context of the above mentioned functionalities of SMCHD1.

Amongst the proteins we validated is RUVBL1, a protein that participates in several protein complexes involved in transcriptional control and chromatin maintenance. RUVBL1 was also identified to putatively bind D4Z4 by enChIP^23^ and we show that reduced RUVBL1 levels, just like the loss of SMCHD1, leads to increased failure of DUX4 repression in FSHD muscle cell cultures. The mild impairment in myogenic differentiation in cells depleted for RUVBL1 and SMCHD1 might be a consequence of the myogenic inhibitory nature and downstream toxicity of DUX4^53,60^.

No other subunits of any of the transcriptional and chromatin modifying multimeric complexes in which RUVBL1 participates (INO80, NuA4, SWR1, TIP60-P400, PAQosome (R2TP) and BAHD1^41–43^) were identified by our SILAC-MS, suggesting that the interaction between SMCHD1 and RUVBL1 may be independent of the described complexes. The BAHD1 repressive chromatin complex, however, contains many subunits that show overlap, or are paralogs of the proteins that form the NuRD complex, which regulates the D4Z4 chromatin structure^23,42^. BAHD1 localizes to Xi in female cells^42,61^, where it assists in recruitment of its complex members such as MIER1^42^. Ectopic expression of BAHD1 in HEK293T cells leads to an ablation of Xi methylation, while simultaneously inducing hypermethylation of autosomal regions such as satellite repeats and interspersed repeats^61^. An exciting hypothesis is the possibility of interplay between components of the BAHD1 repressive chromatin complex and SMCHD1 to maintain proper Xi silencing or repeat repression.

We also showed that SMCHD1 interacts with the Cohesin component RAD21. Cohesin is a chromatin binding complex, which besides RAD21 consists of the SMC1/SMC3 heterodimer and accessory proteins^62^. Unlike SMC1/SMC3, SMCHD1 forms homodimers and has not been described to interact with other SMC family members^63^. Previous studies have shown that RAD21 and SMC3 are dispensable for *DUX4* silencing in somatic cells^13^, although they are present at D4Z4^23,48^. Whether the interaction of RAD21 and SMCHD1 is direct and spatiotemporally regulated, or whether SMC1 and/or SMC3 are involved in the formation of this complex has not been investigated. Neither is it known if any of these complex components or their accessory proteins are required for each other’s recruitment to D4Z4.

We validated the interaction of SMCHD1 with the recently characterized protein EZHIP (CXorf67). This interaction is intriguing as overexpression of EZHIP leads to inhibition of the PRC2 protein EZH2, and loss of the repressive histone modification H3K27me3^44,46^, a modification that is enriched at D4Z4 in FSHD2 patients^13^. This loss of H3K27me3 upon ectopic expression of EZHIP in HEK293T cells^44,46^ was also observed in our hTERT-RPE1 experiments. However, EZHIP is not expressed in muscle cells, excluding a role in the maintenance of DUX4 repression in skeletal muscle. The presence of Ezhip in the earliest stages of mouse embryogenesis, however, at the time that Dux also peaks, warrants further investigation into the role of EZHIP in chromatin remodelling considering the highly dynamic regulation of H3K27me3 at this stage of development^64^.

EZHIP has been described as a MBDT1 fusion protein in endometrial stromal sarcoma^50^. The U2OS cell line in which we performed our SILAC-MS screen is derived from an osteogenic sarcoma^65^. Western blotting identified an intense EZHIP band at a higher molecular weight than expected for the protein, which may also be the result of an EZHIP fusion. Previous EZHIP research used U2OS cells and showed EZHIP at a higher molecular weight^45^, although none of the studies referred to the irregularity of U2OS-derived EZHIP^45,47,66^. Nevertheless, it has EZH2 inhibiting properties, as knockdown of EZHIP in U2OS cells by siRNA rescues H3K27me3 levels, and localizes to the nucleus suggesting it to be biologically active.

We also investigated the potential interaction of SMCHD1 with mediator of DNA damage checkpoint 1 (MDC1), as recently SMCHD1 has been described as a factor contributing to telomere maintenance by the DNA damage repair (DDR) machinery through facilitating ATM signalling^57^. MDC1 is a known component of the DDR signalling required for detection and repair of damaged telomeres, inducing non-homologous end-joining (NHEJ)^67^. Due to MDC1 antibody cross-reactivity with GFP-SMCHD1 we were unable to confirm this interaction (data not shown).

Since SMCHD1 was recently reported to be involved in de novo D4Z4 DNA methylation in human pluripotent cells^15^ and in the termination of the first zygotic genome activation in mouse 2-cell embryos by Dux repression^56^, we bioinformatically explored the co-expression of the proteins identified by SILAC-MS during early mouse embryogenesis and postnatal human muscle cell differentiation. Apart from the EZHIP interaction, several SMCHD1 protein interactors show overlapping expression patterns in early mouse embryogenesis (*Ruvbl1*, *Ruvbl2*, *Mdc1* and *Hcfc1*) or human postnatal muscle differentiation (*SMCHD1*, *RUVBL1*, *HNRPA1*, *HCFC1* and *PRPF8*), providing additional evidence for their co-existence and potential cooperation in chromatin modelling and gene silencing. The downward trend in expression of some of these genes during myogenesis might make muscle cells more susceptible to DUX4 expression.

A notable absentee in our list of identified SMCHD1 interactors is the protein Ligand dependent Receptor Interacting Factor 1 (LRIF1). LRIF1 is one of the first interaction partners described for SMCHD1^49^, was detected in a previous Smchd1 interactome screen in mouse ES cells^63^, and binds to the D4Z4 repeat where it is involved in the repression of DUX4^11^. Its absence in our study can be explained by the observation that LRIF1 is not expressed in U2OS cells, which we used for the SILAC-MS analysis. Indeed, the interaction between both proteins could be readily confirmed in HEK293T and HeLa cells. The limited overlap between our dataset and the Brideau dataset can probably be explained by the use of different model organisms, cell type and purification approach^63^.

In conclusion, we here present the protein interactome of the chromatin repressor SMCHD1. We confirm a number of proteins and studied their involvement in the D4Z4 chromatin structure and repression of DUX4 in muscle cells. We showed that knocking down RUVBL1 in FSHD muscle cells further derepresses DUX4 and provided evidence that some of these proteins may act with SMCHD1 at different genomic sites in a spatiotemporal context, suggesting that SMCHD1 may participate in different chromatin complexes. This putative SMCHD1 protein interactome may serve as a guide into SMCHD1 chromatin function.

## Supplementary Information

Supplementary Information 1.

Supplementary Information 2.

Supplementary Information 3.

Supplementary Information 4.
