# Supplementary material for: A proteomics study identifying interactors of the FSHD2 gene product SMCHD1 reveals RUVBL1-dependent DUX4 repression
Source: Sci Rep. 2021 Dec 8;11:23642. doi: 10.1038/s41598-021-03030-3 (PMC8654949; doi:10.1038/s41598-021-03030-3)
Supplement: Supplementary file 1 — Supplementary Information 1. [file 41598_2021_3030_MOESM1_ESM.pdf]

Supp. Figure 1: Characterization of stable GFP-SMCHD1 U2OS clones and dataset analysis using the Enrichr web tool of 47 nuclear SMCHD1 interactors identified by SILAC-MS with a median H/L ratio >1.5.

A: Western blot analysis of six single cell U2OS clones expressing GFP-SMCHD1. Absence of a 250 kDa band in the GFP channel indicates defective construct integration. Clones used for SILAC-MS are indicated in red. Clones excluded from use are indicated in light grey.

B: RT-qPCR analysis of *GFP-SMCHD1* and *eGFP* expression levels in the clones presented in A.

C: Widefield fluorescent microscopy analysis of GFP-SMCHD1 expressing clones presented in A. Clones B3, B6 and G6 were excluded due to cytoplasmic GFP signal.

D: Cellular fractionation of wildtype U2OS cells, cytoplasmic GFP-SMCHD1 clone B6 and nuclear GFP-SMCHD1 (functional) clone A7 in two concentrations of input material as indicated. Tubulin and H3 were used as markers of the cytoplasmic and chromatin fraction, respectively.

E: Validation western blot of a fraction of two of the GFP-IP samples used for MS-analysis shown in figure 1. Tubulin and actin are used as cytoplasmic protein markers, while H3 is used as a chromatin marker.

Supp. Figure 2: Individual plots of each replicate of SILAC-MS.

For each replicate experiment, Log2 transformed values for forward and reverse (Heavy and light media) were plotted together as indicated for each of the clonal lines. Specific protein labels and colours of dots are the same as figure 1B.

Supp. Figure 3: Validation of SMCHD1 interaction partners.

A: Western blot analysis of endogenous RUVBL1 co-immunoprecipitation after purification of GFP-SMCHD1 expressed in the two stable U2OS clones used for the SILAC-MS.

B: Western blot analysis after GFP-IP or HA-IP in HEK293T, U2OS and HeLa cells overexpressing GFP-SMCHD1 or 3xHA-SMCHD1 as indicated. U2OS cells lack the known SMCHD1 interacting protein LRIF1, which is only co-purified in HEK293T and HeLa cells. RUVBL1 is co-purified in all cell types. A selection of panels is also shown in figure 2A.

C: Western blot after immunoprecipitation of ectopically expressed GFP-SMCHD1 in HEK293T cells shows co-precipitation of endogenous RUVBL2. Outlines in the composite figure panels denote parts of the membrane originating from the same western blot membrane.

D: Western blot after immunoprecipitation of ectopically expressed GFP-SMCHD1 in U2OS cells shows co-precipitation of endogenous RUVBL2. Outlines in the composite figure panels denote parts of the membrane originating from the same western blot membrane.

E: Western blot after immunoprecipitation of endogenous EZHIP in HEK293T, U2OS and HeLa cells. Upon purification of EZHIP, endogenous SMCHD1 can be detected in the IP fraction for each cell type. The asterisk denotes the presence of IgG heavy chains present in the sample, which partially obscure the ~50 kDa predicted EZHIP signal. Arrowhead indicates a higher molecular weight EZHIP band, specific for U2OS cells.

Supp. Figure 4: Characterization of EZHIP (CXorf67).

A: Confocal immunofluorescent analysis of hTERT-RPE1 cells overexpressing HA-EZHIP and stained for nuclei (DAPI), HA-tag and H3K27me3. Scalebar: 25 µm.

B: Quantification of the presence of H3K27me3 signal in hTERT-RPE1 cells. Shown are pooled counts of 120 cells imaged in experiments as exemplified in A. Cells were ectopically expressing HA-EZHIP, GFP-EZHIP or mCherry-EZHIP (22 counted) or not expressing tagged-EZHIP (98 counted). When cells overexpress EZHIP, 77.3% of cells show a low H3K27me3 signal, compared to 23.5% of cells not expressing exogenous EZHIP.

C: Confocal immunofluorescent analysis of U2OS cells transfected with a non-targeting siRNA (siNT) or siRNAs targeting EZHIP as indicated. Cells were stained for nuclei (DAPI), EZHIP and H3K27me3. Scalebar: 10 µm.

D: Western blot analysis of the expression of EZHIP in various cell lines. Expected size: ~52 kDa. The arrowheads indicate a higher molecular weight EZHIP band, specific for U2OS cells.

E: RT-qPCR analysis of the expression of *EZHIP* in various cell lines normalized to *GUSB*. ND: Not reliably detected.

E: Stimulated emission depletion (STED) super resolution microscopy analysis of endogenous EZHIP in U2OS cells. Scalebar: 2.5 µm.

Supp. Figure 5: Gene expression analysis in primary myocytes. RT-qPCR analysis of primary myoblast (MB) and myotube (MT) samples derived from healthy controls, FSHD1 individuals and FSHD2 individuals, allowing assessment of expression differences induced by myogenic differentiation and/or FSHD status. Expression of *RUVBL1, RUVBL2, MYOG, MYH3, SMCHD1, DUX4, ZSCAN4* and *KHDC1L* are shown for all samples. *MBD3L2, TRIM43* and *GAPDH* are shown for a subset of control, FSHD1 and FSHD2 samples. *GAPDH* is shown for assessment of stability of *GUSB* normalization.

Supp. Figure 6: Effect of RUVBL1 knockdown in FSHD myocytes.

A: Additional RT-qPCR analysis of FSHD derived myoblast samples shown in figure 3B knocked down for *RUVBL1* with shRNAs. Expression of *SMCHD1, MYH3, KHDC1L, MBD3L2, TRIM43* and *RUVBL2* are shown. Error bars: SEM. (*: P value <0.05, **: P value <0.01, NS: Not-Significant – Kruskal-Wallis One-Way ANOVA)

B: Representative western blot analysis of myotube samples depleted for RUVBL1 and SMCHD1 as indicated. Corresponding microscopy and chromatin samples were used in figures 3C, 3D, 3E and 3F.

C: Example of output of high content analysis for DUX4 positive myonuclei. Original images on the left panels are overlaid in the right panels with a grid indicating DUX4 negative nuclei (blue dots) or DUX4 positive nuclei (red dots).

D: Separate violin plots of each replicate of high content analysis for DUX4 positive nuclei of slides shown in 3C.

E: Separate box and whisker plots of each replicate of myogenic fusion index determined in slides from 3C by high content analysis.

Supp. Figure 7: Analysis of RNA-seq datasets of murine early development. Fragments Per Kilobase Million (FPKM) are plotted on the Y-axis, while developmental stage is indicated on the X-axis. Data originally published by Wu et al. 2016.

A: Overlay of expression of *Dux* (Red), *Smchd1* (Green) and *Ezhip* (Blue) in early development. Separate graphs are provided for: B: *Ezhip*; C: *Smchd1*; D: *Dux*; E: *Ruvbl1*; F: *Ruvbl2*; G: *Mdc1*; H: *Hcfc1*; I: *Myo1c*;

J: *Zscan4a*; K: *Zscan4b*; L: *Hnrnpa0*; M: *Hnrnpa1*; N: *Prpf8*; O: *Trim22 (not present in mouse: N/A)*; P: *Ndufa9*; Q: *Rad21*; R: *Sh3pxd2b*; S: *Rbmx*; T: *Dis3* and U: *Hspa1a*.

Supp. Figure 8: Analysis of RNA-seq by Yao et al. of genes of interest in cultured myocytes. Expression of myoblasts and myotubes from both controls and FSHD individuals are plotted as indicated. N/A: Data for gene not available in dataset.

Supp. Figure 9: Analysis of RNA-seq by Yao et al. of genes of interest in muscle biopsies. Expression from both controls and FSHD individuals are plotted as indicated. N/A: Data for gene not available in dataset.

Supp. Figure 10: Analysis of RNA-seq by Trapnell et al. of genes of interest in differentiating myocytes. Expression of genes at various days of myogenic differentiation is plotted as indicated.

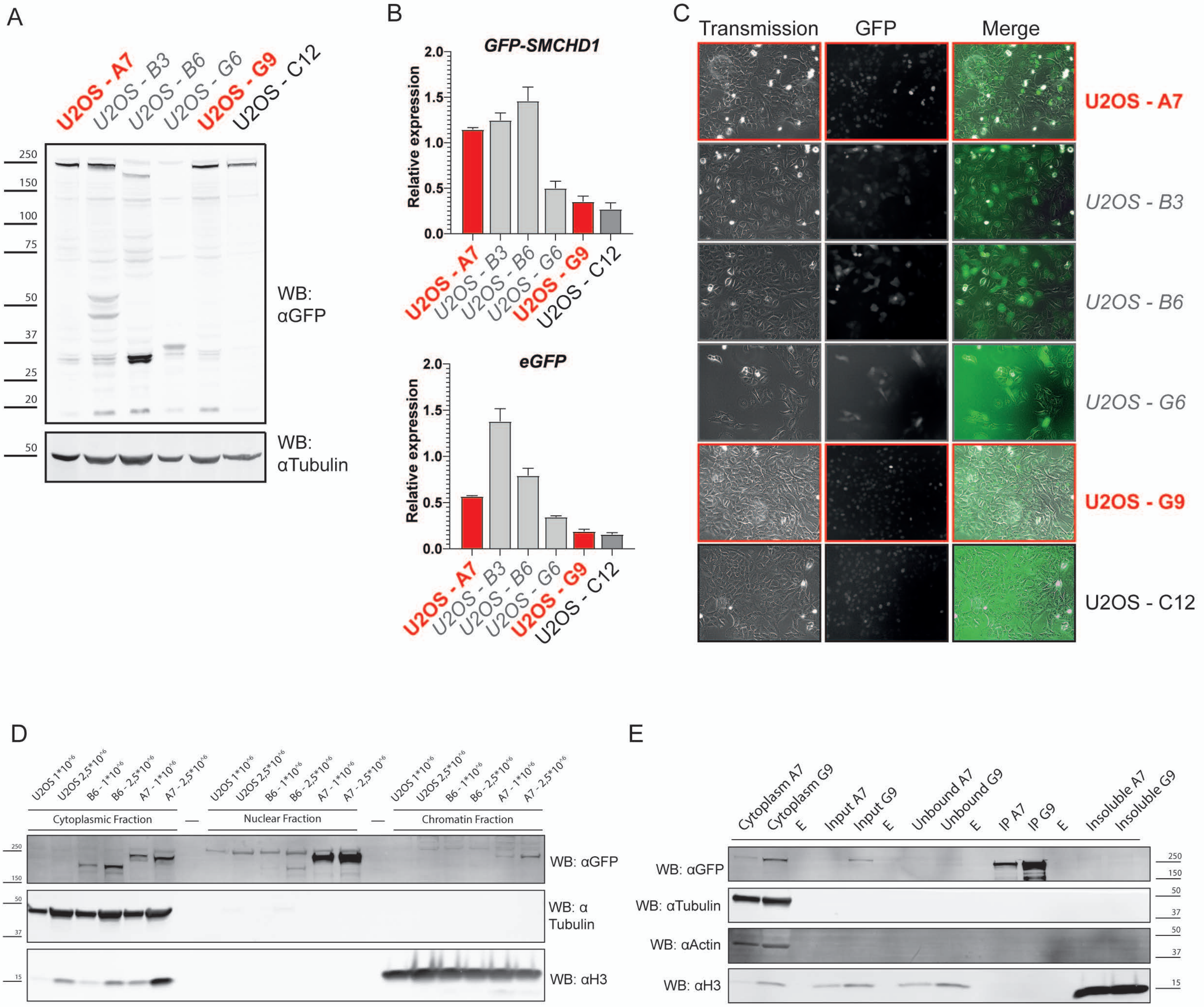

Supp. Figure 1

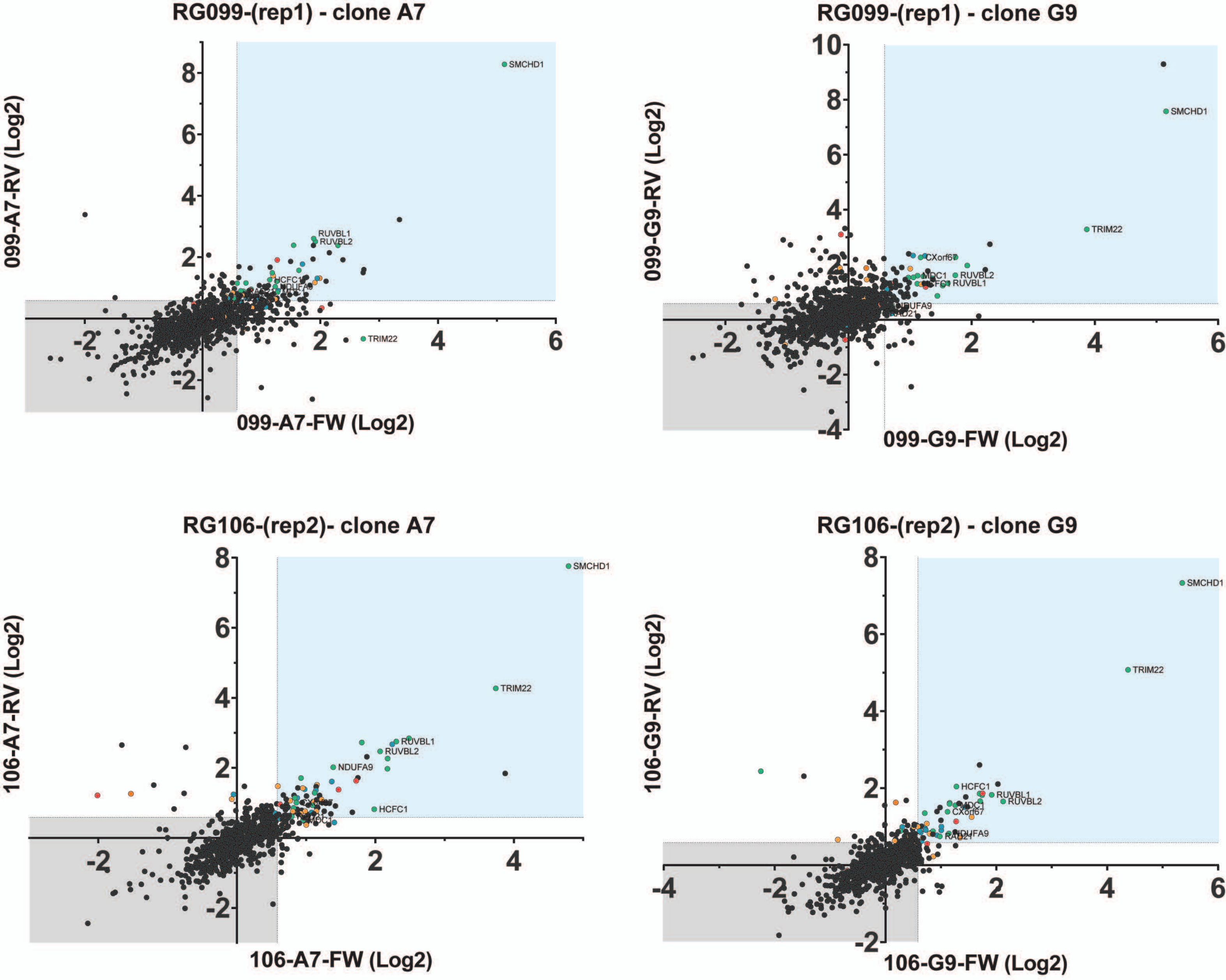

Figure 2 Supplementary

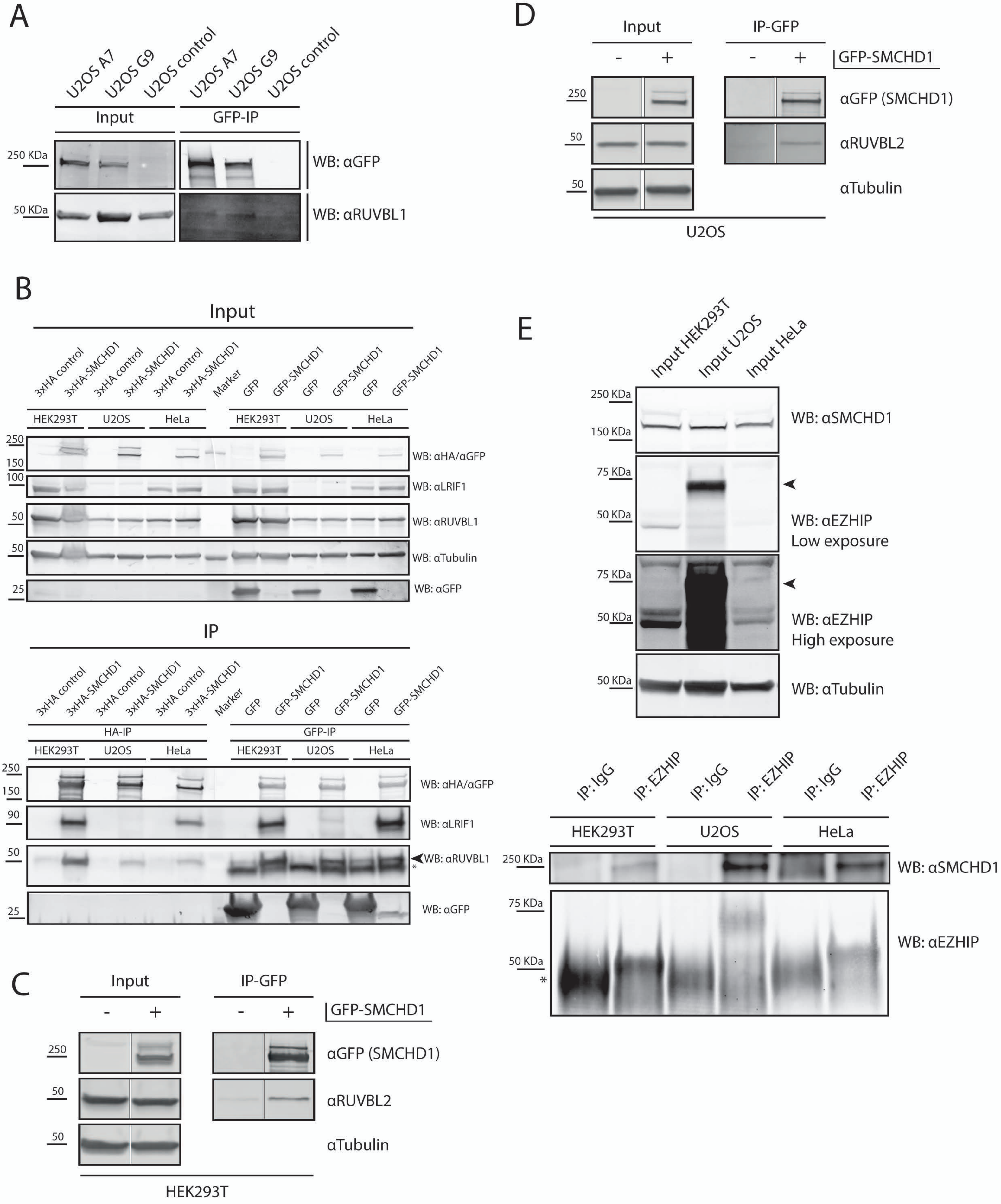

Figure 3 Supplementary

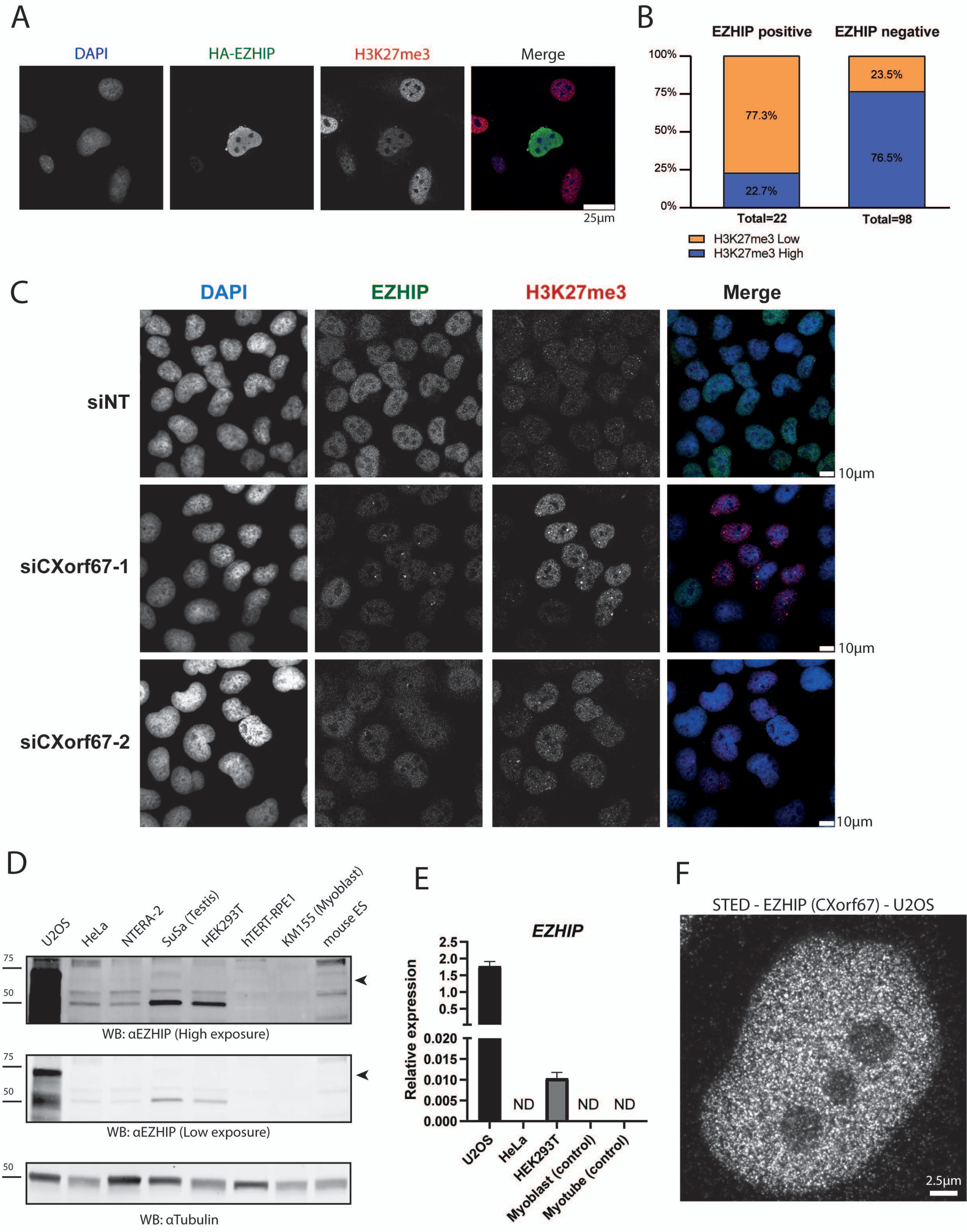

Figure 4 Supplementary

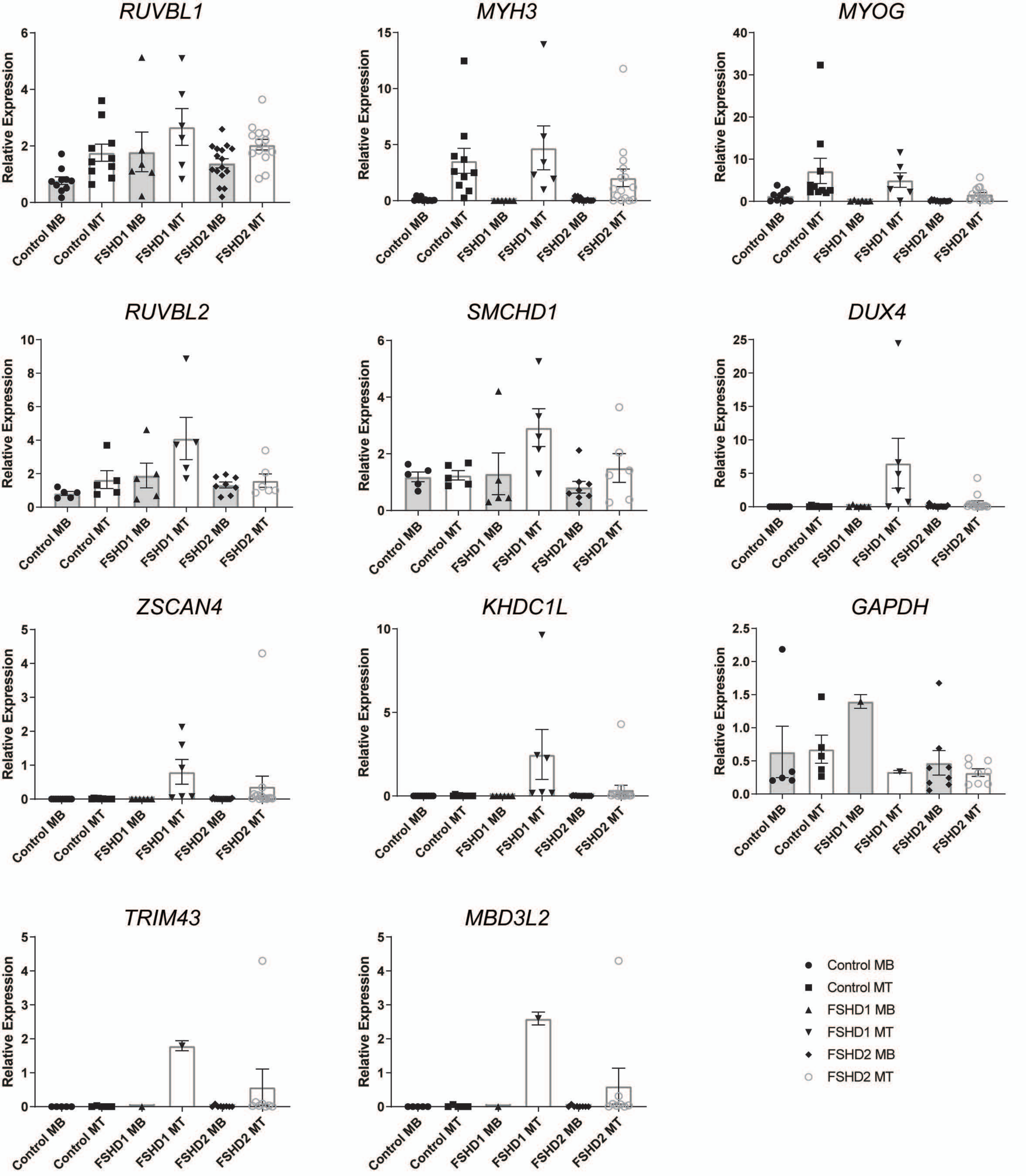

Figure 5 Supplementary

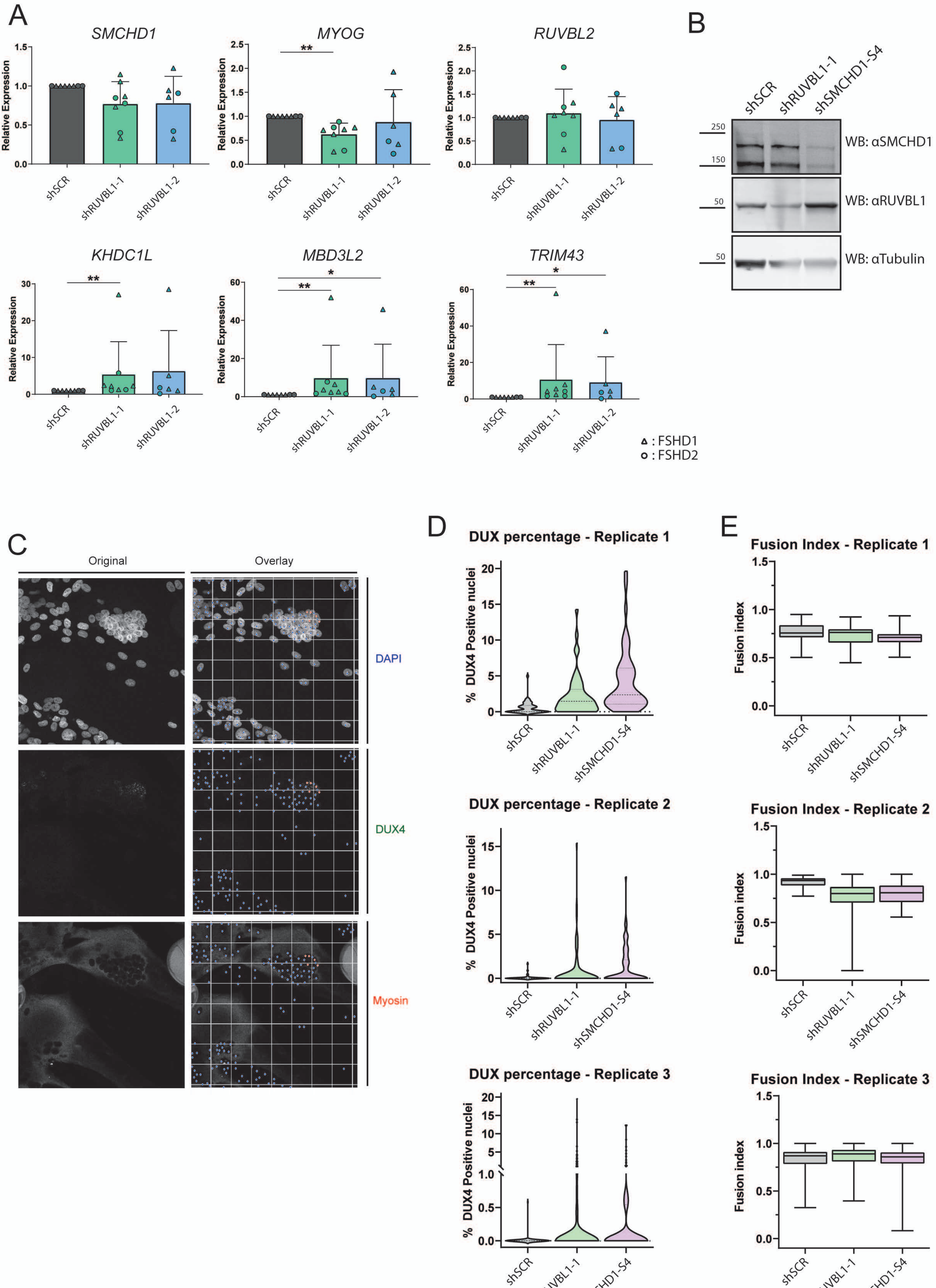

Supp. Figure 6

Figure 7 Supplementary

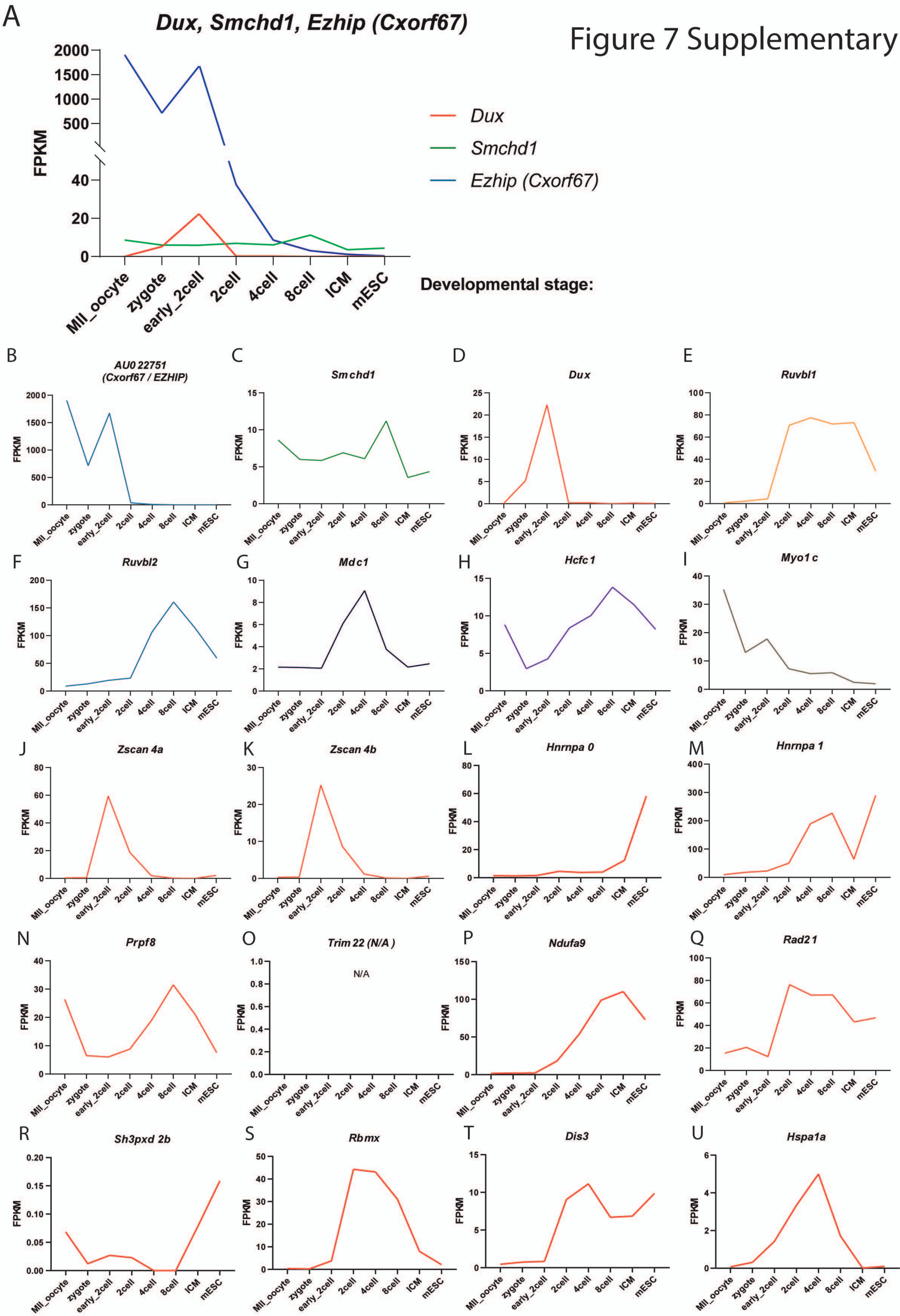

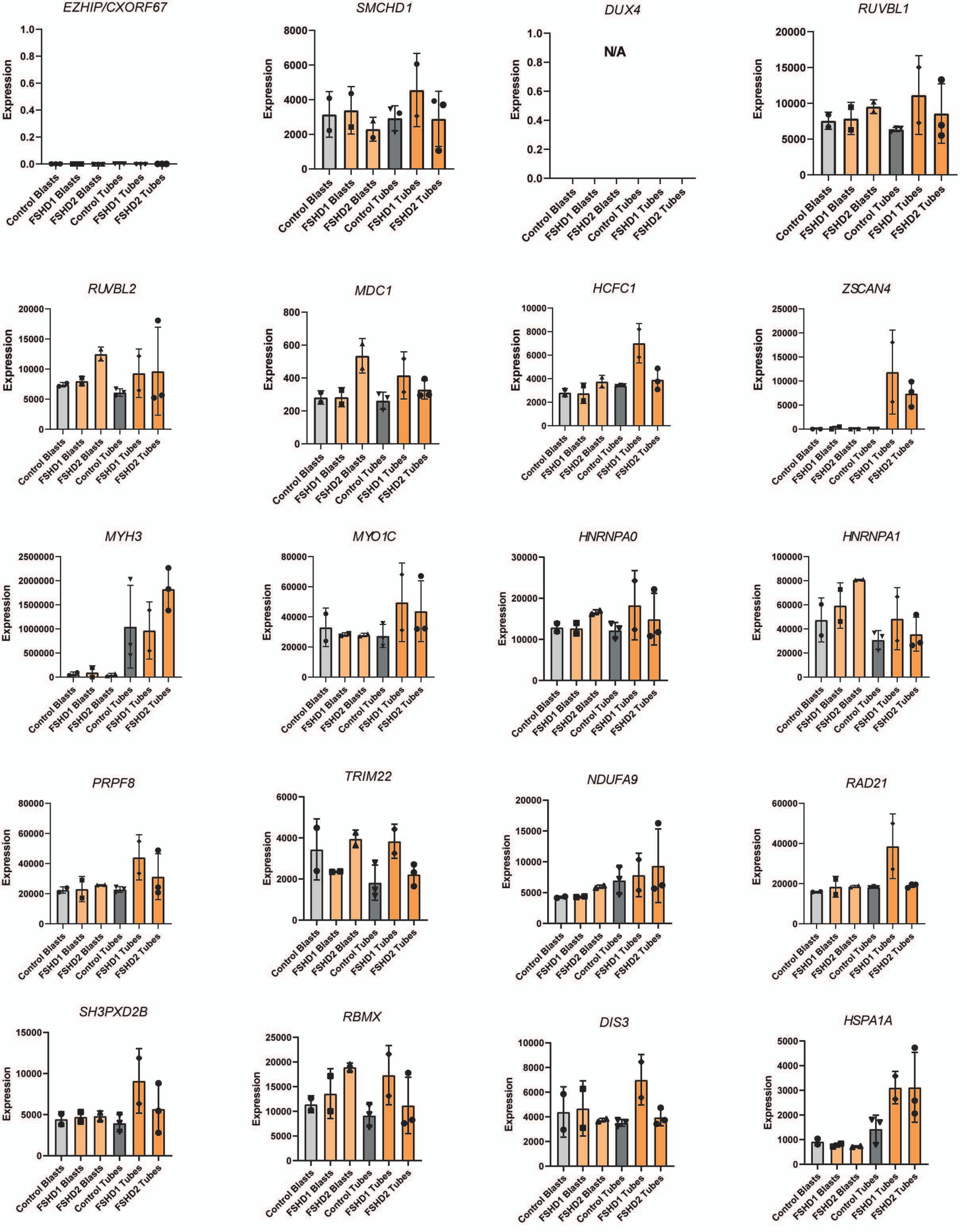

Cultured genes Yao et al. 2014
N/A: Data not available

Figure 8 Supplementary

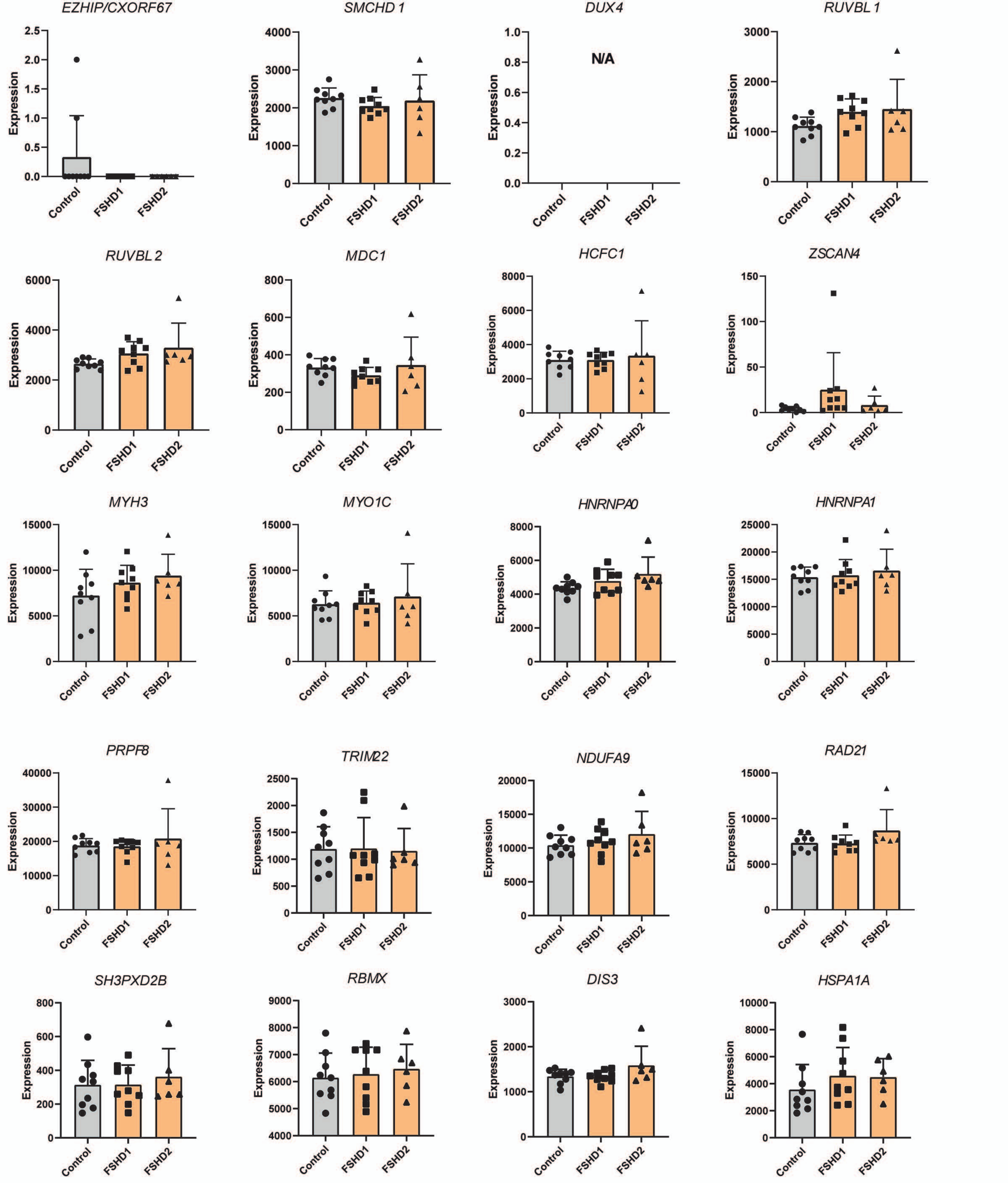

Biopsy genes Yao et al. 2014

N/A: Data not available

Figure 9 Supplementary

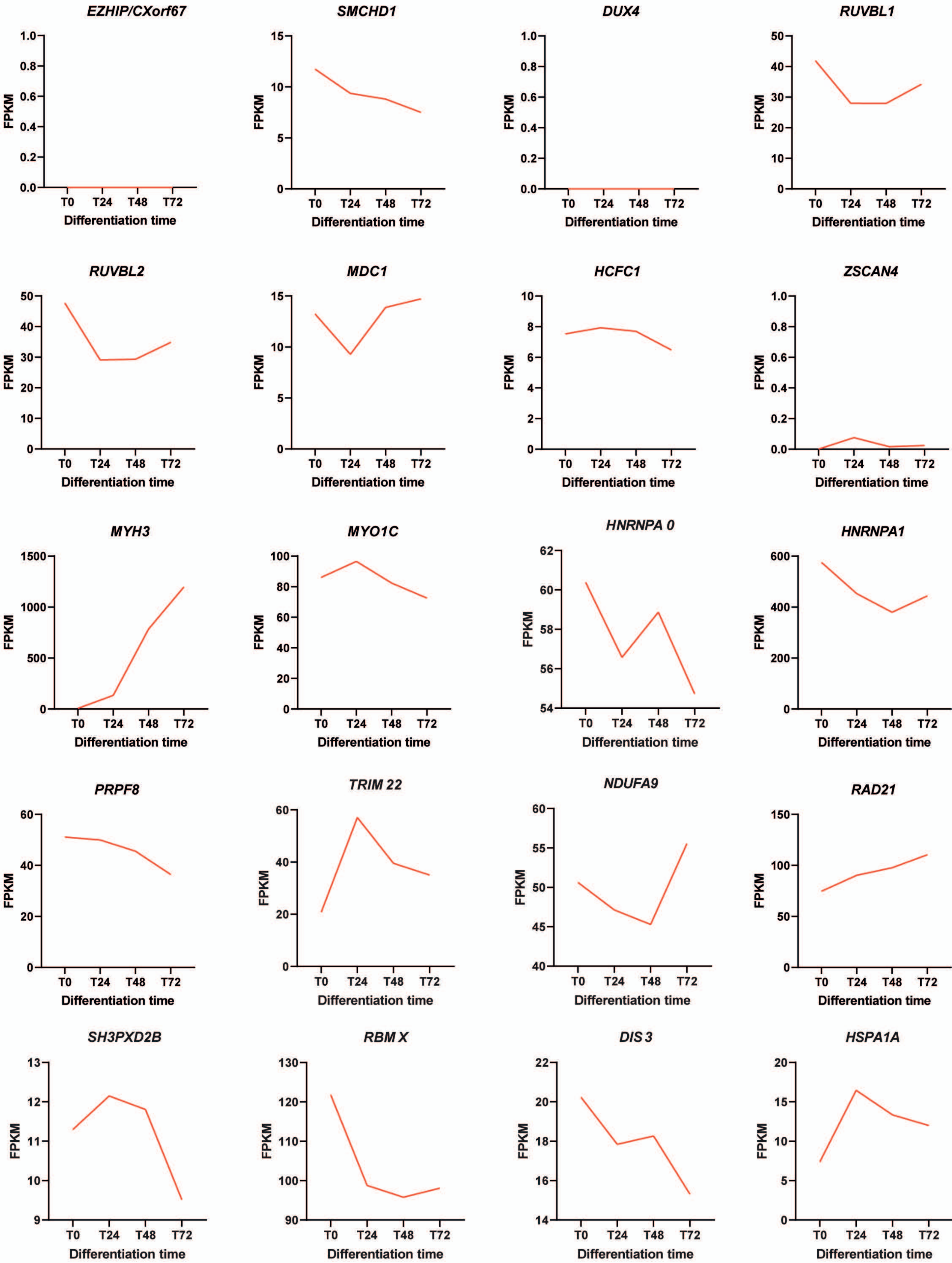

Myogenic differentiation - Trapnell et al. 2014

Figure 10 Supplementary
