# Supplementary material for: A proteomics study identifying interactors of the FSHD2 gene product SMCHD1 reveals RUVBL1-dependent DUX4 repression
Source: Sci Rep. 2021 Dec 8;11:23642. doi: 10.1038/s41598-021-03030-3 (PMC8654949; doi:10.1038/s41598-021-03030-3)
Supplement: Supplementary file 2 — Supplementary Information 2. [file 41598_2021_3030_MOESM2_ESM.pdf]

Figure 2A

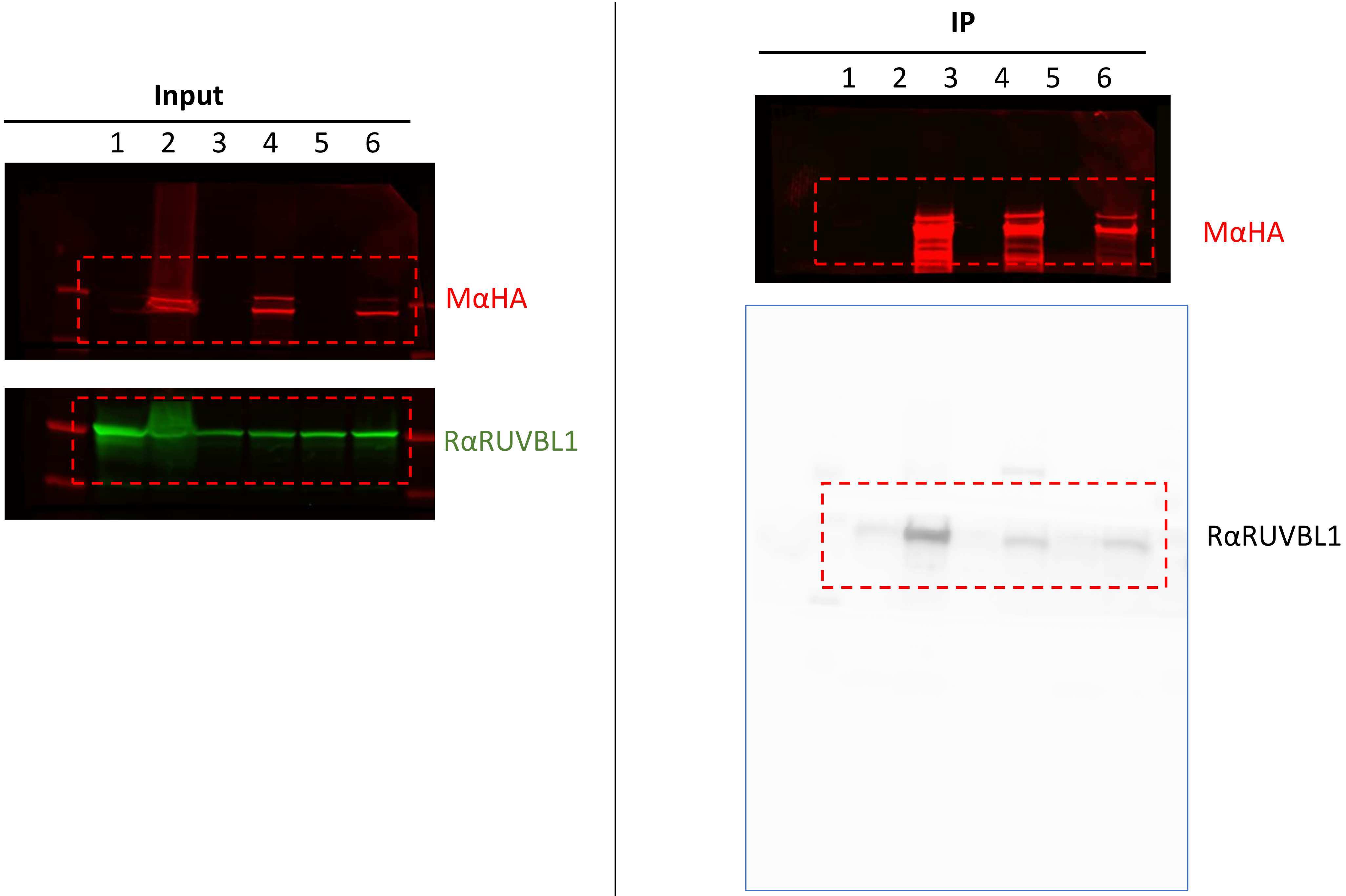

Goossens et. al.

Figure 2B

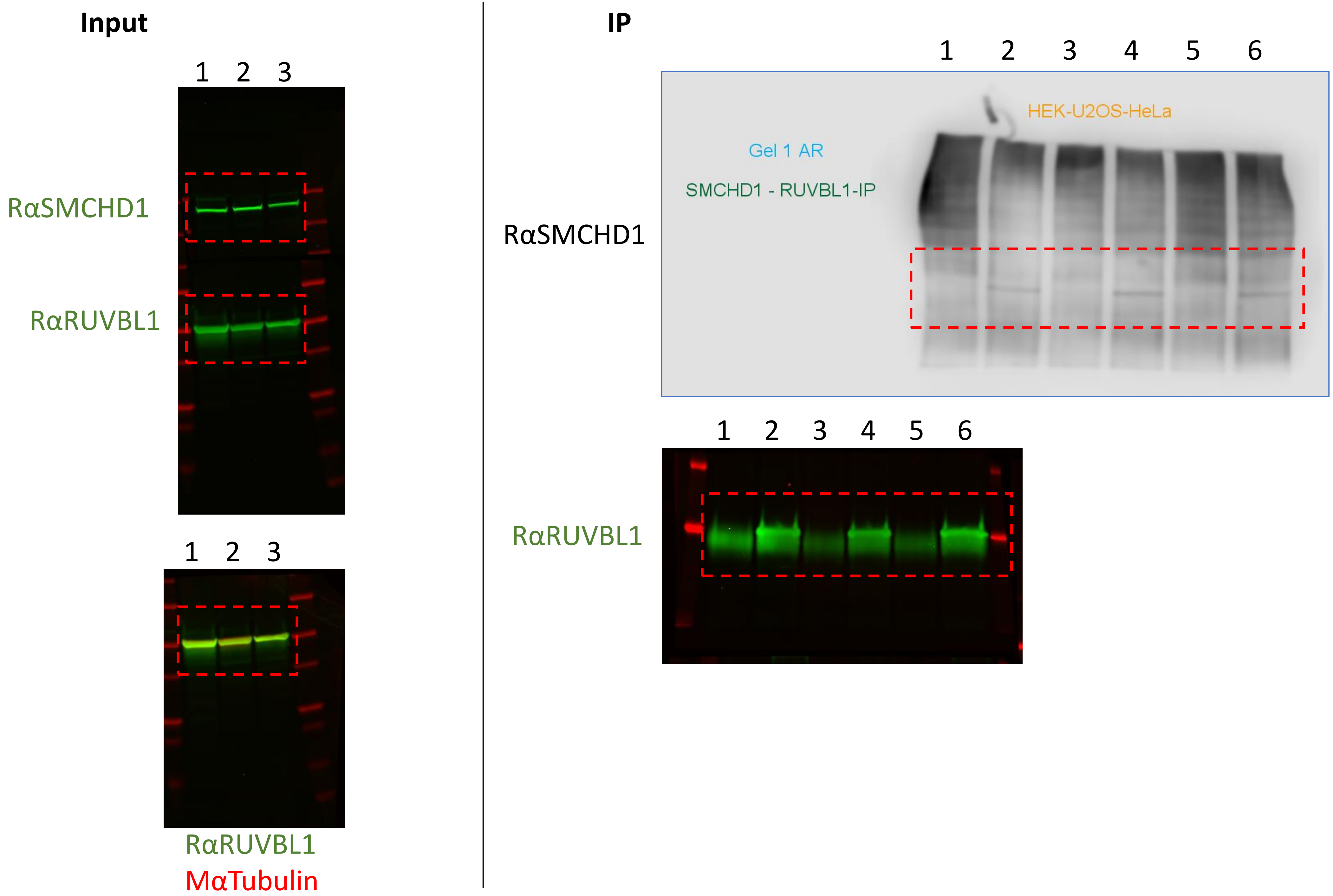

Goossens et. al.

Figure 2C

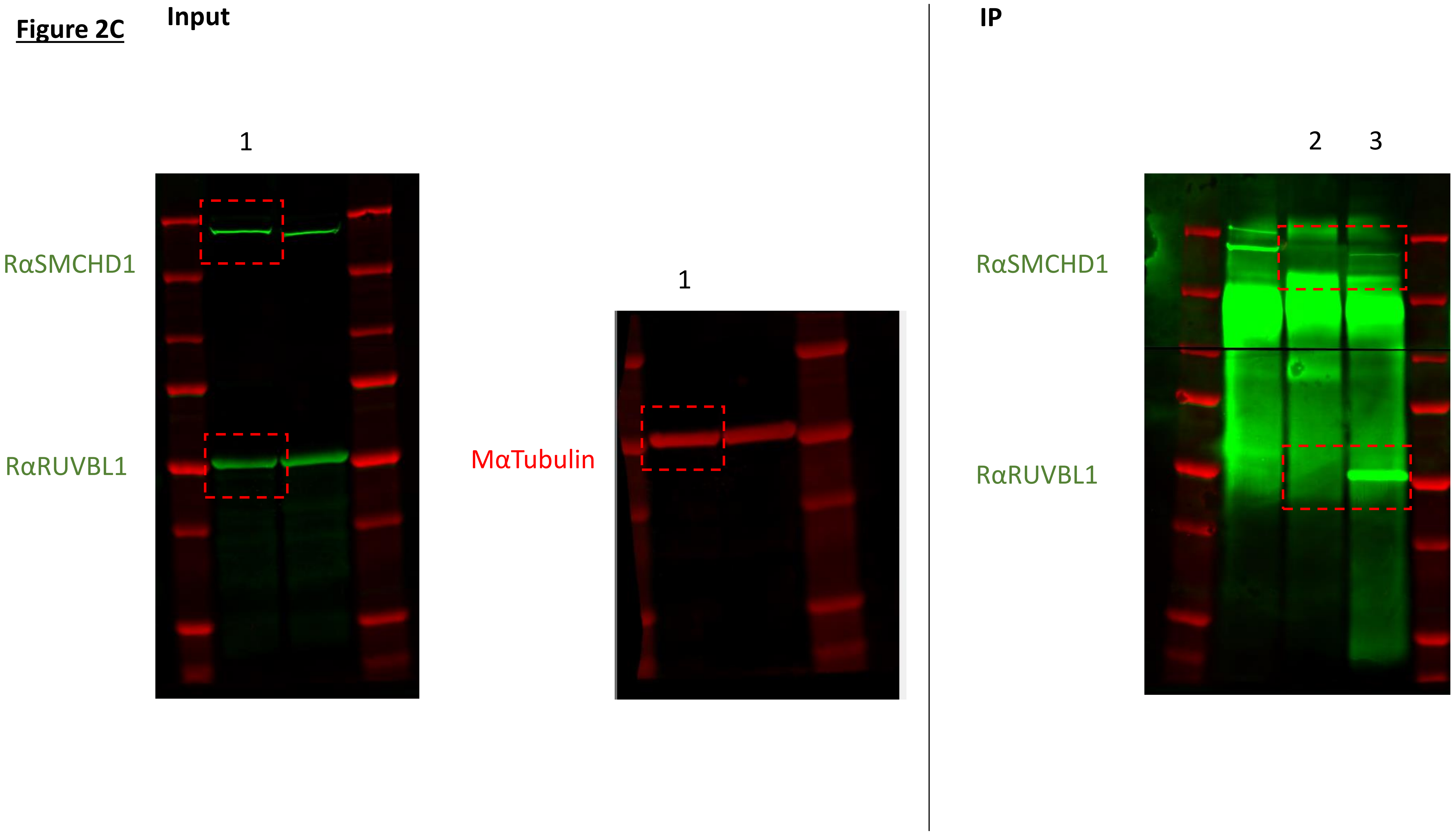
Input
IP
1
1
2
3
RαSMCHD1
RαRUVBL1
MαTubulin
RαSMCHD1
RαRUVBL1

Figure 2D

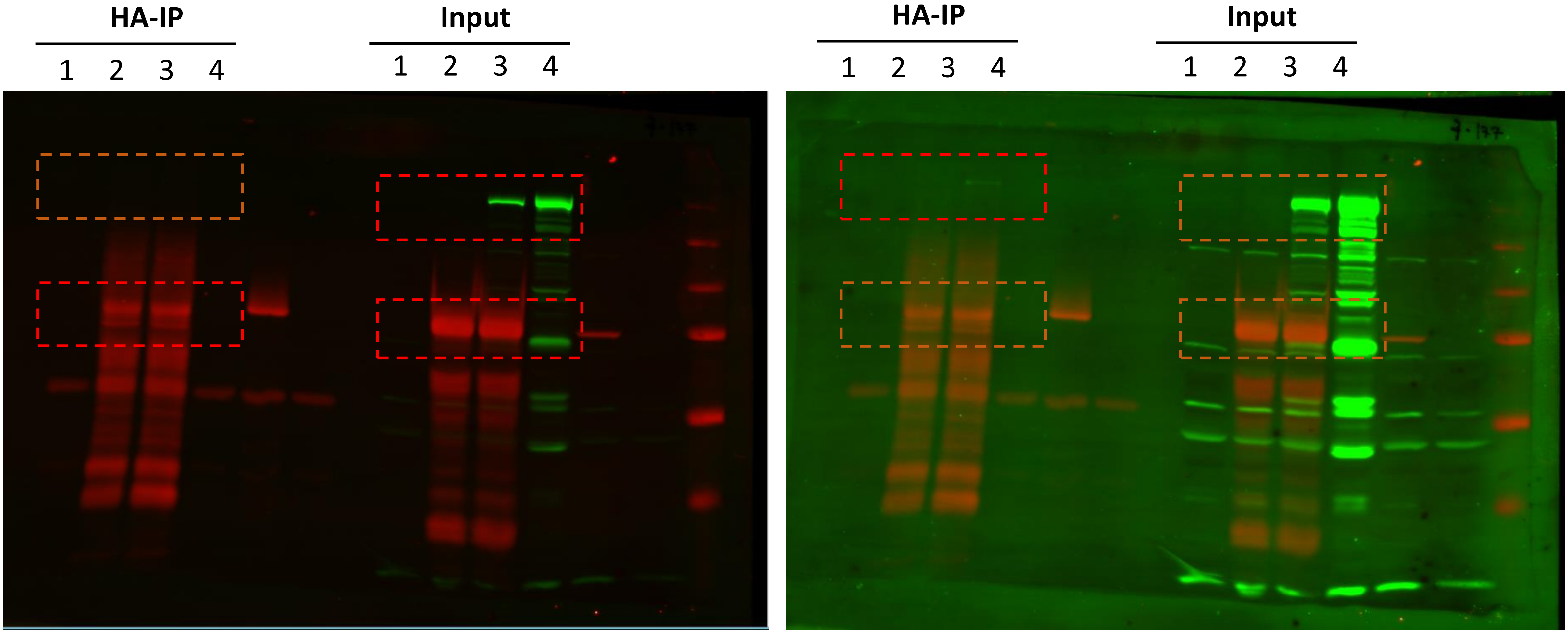

Low exposure

RαFlag -- MαHA

High exposure

RαFlag -- MαHA

Goossens et. al.

Figure 2E

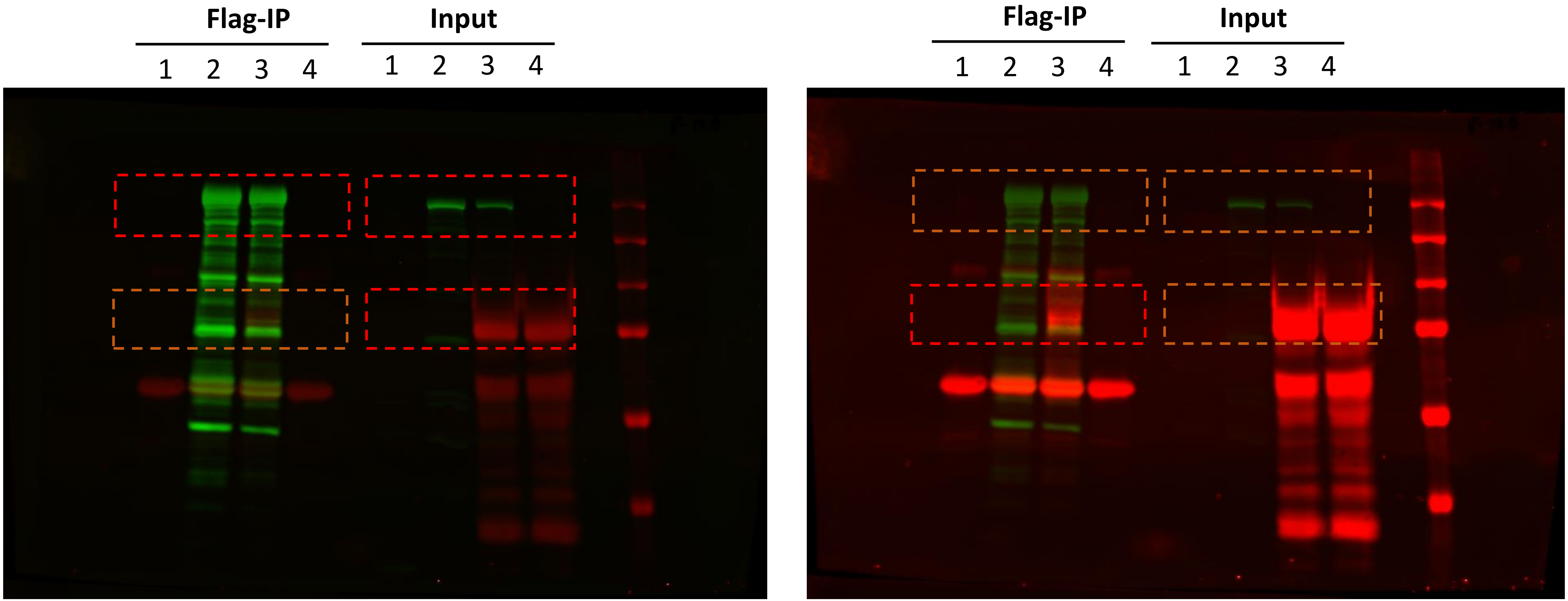

Low exposure

High exposure

RαFlag -- MαHA

RαFlag -- MαHA

Goossens et. al.

Figure 2F

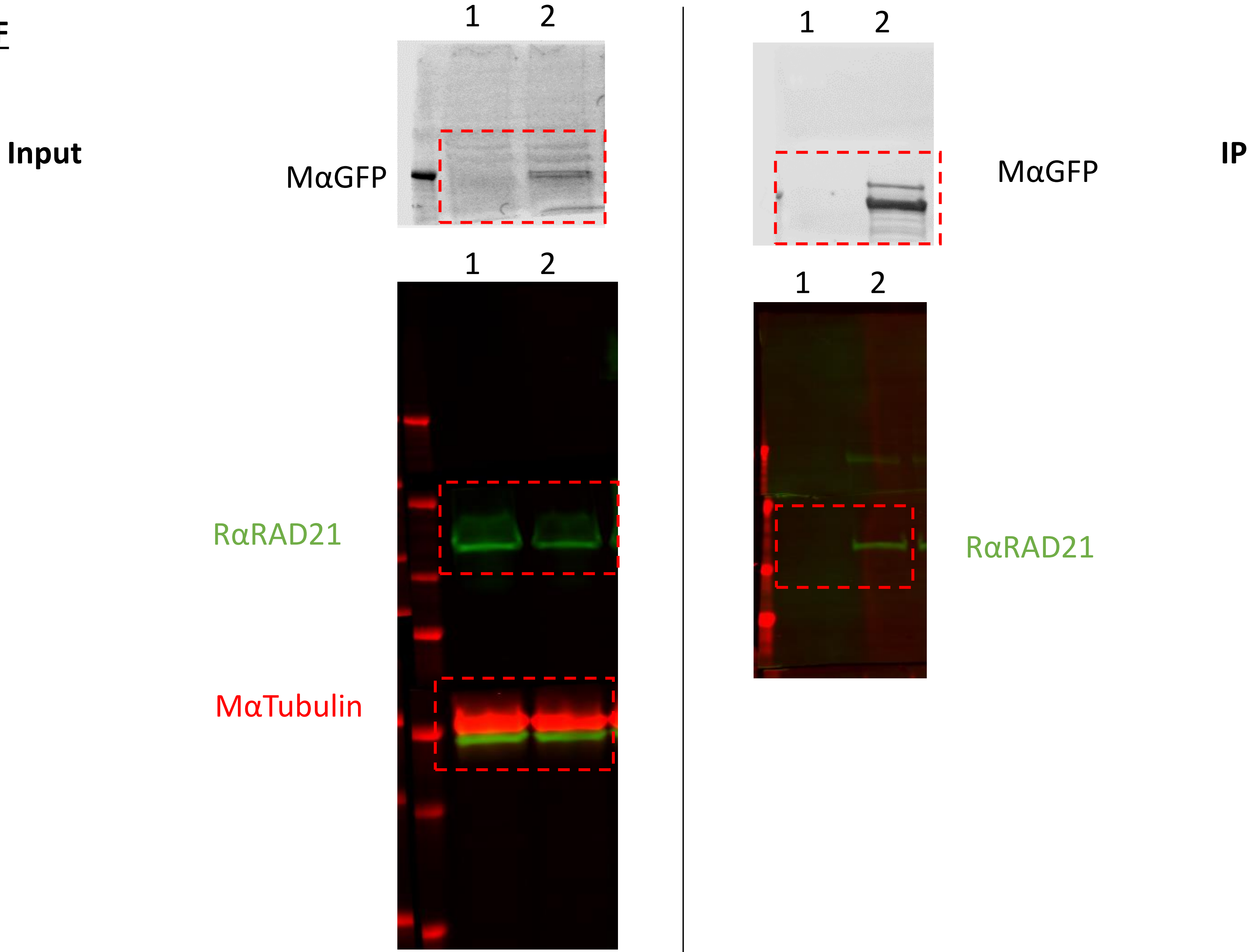

Goossens et. al.

Figure 3A

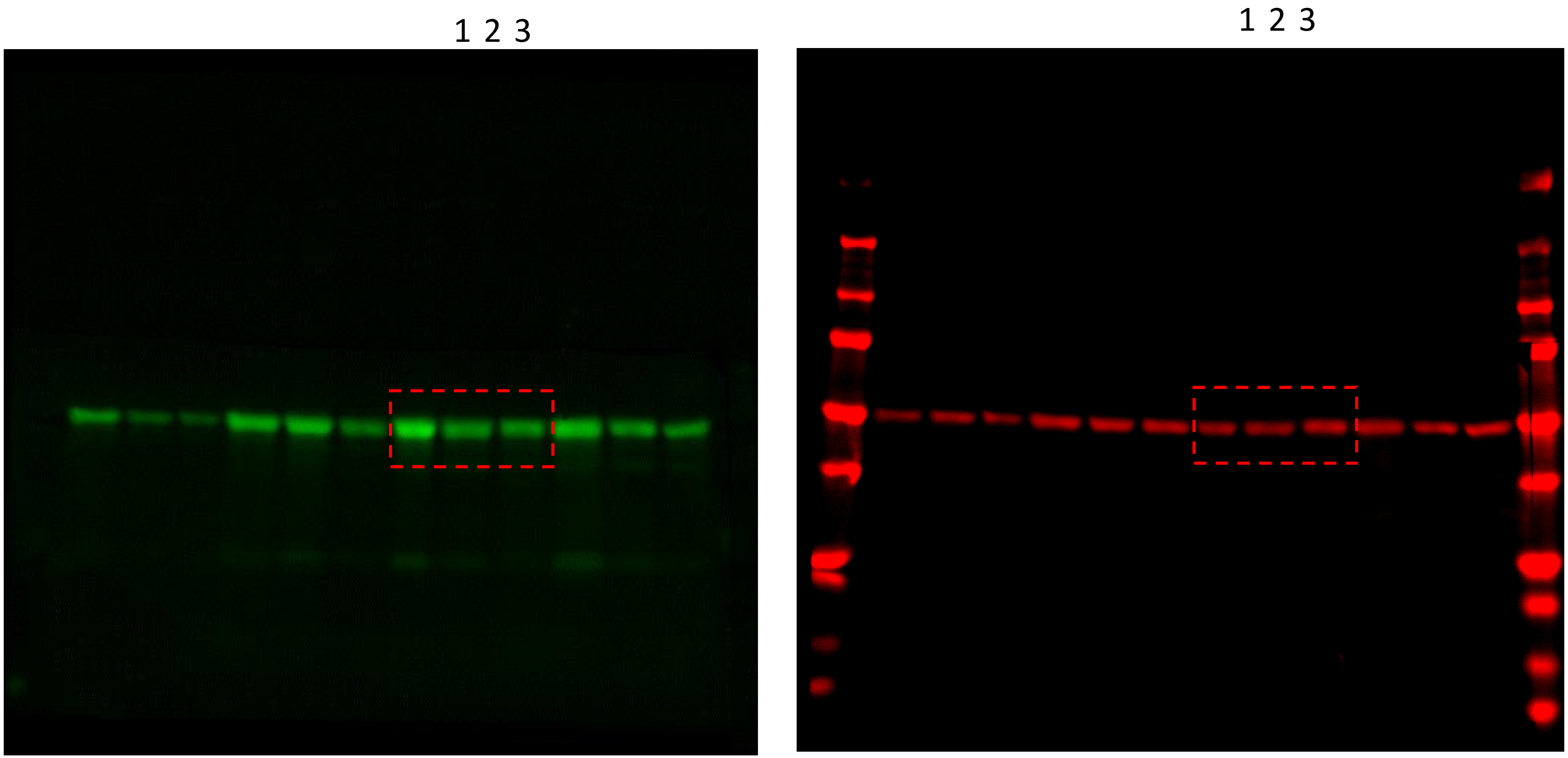

Channel 800nm (RUVBL1)

Channel 680nm (Tubulin)

Goossens et. al.

Supp. Figure 1A

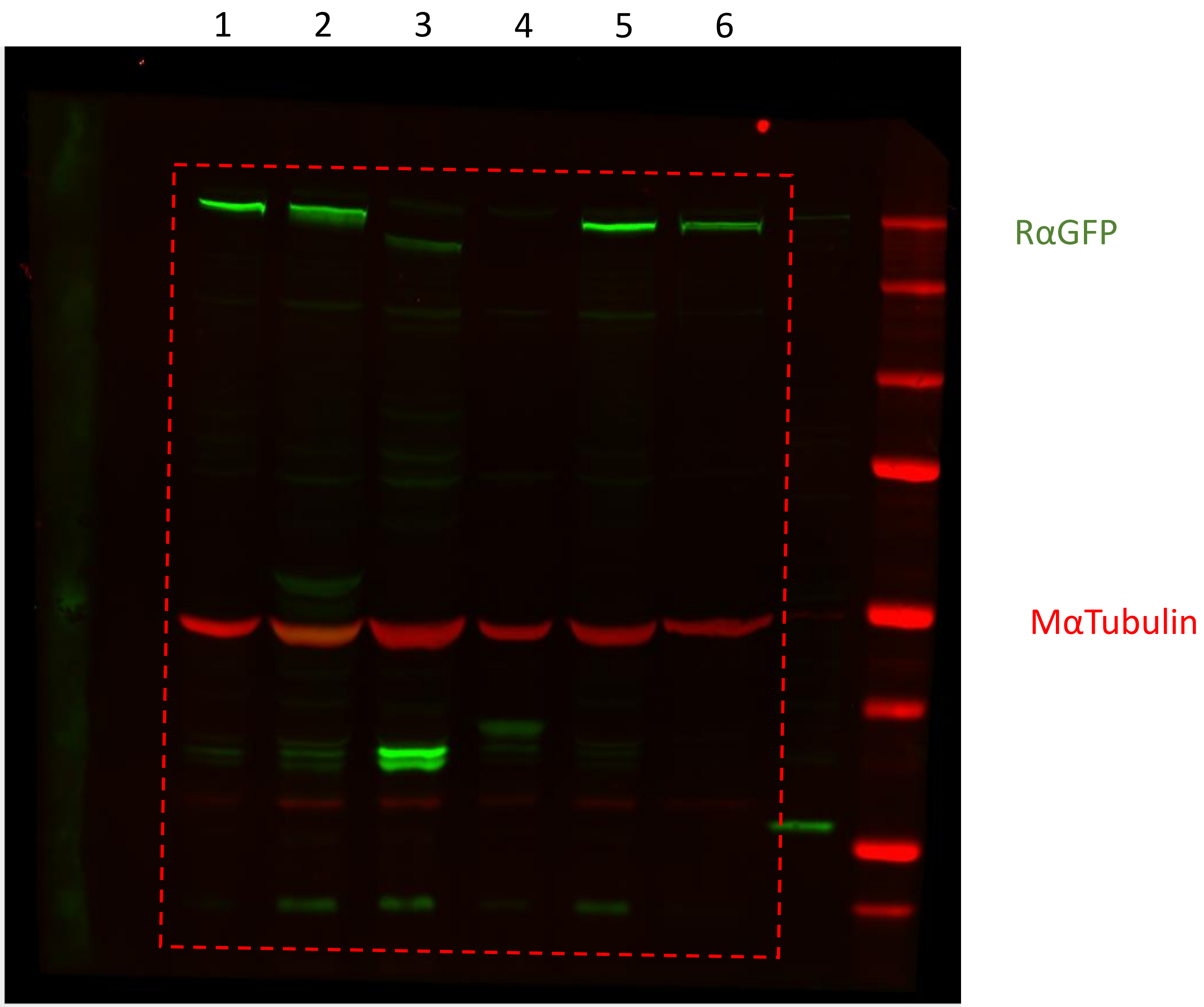

Goossens et. al.

Supp. Figure 1D

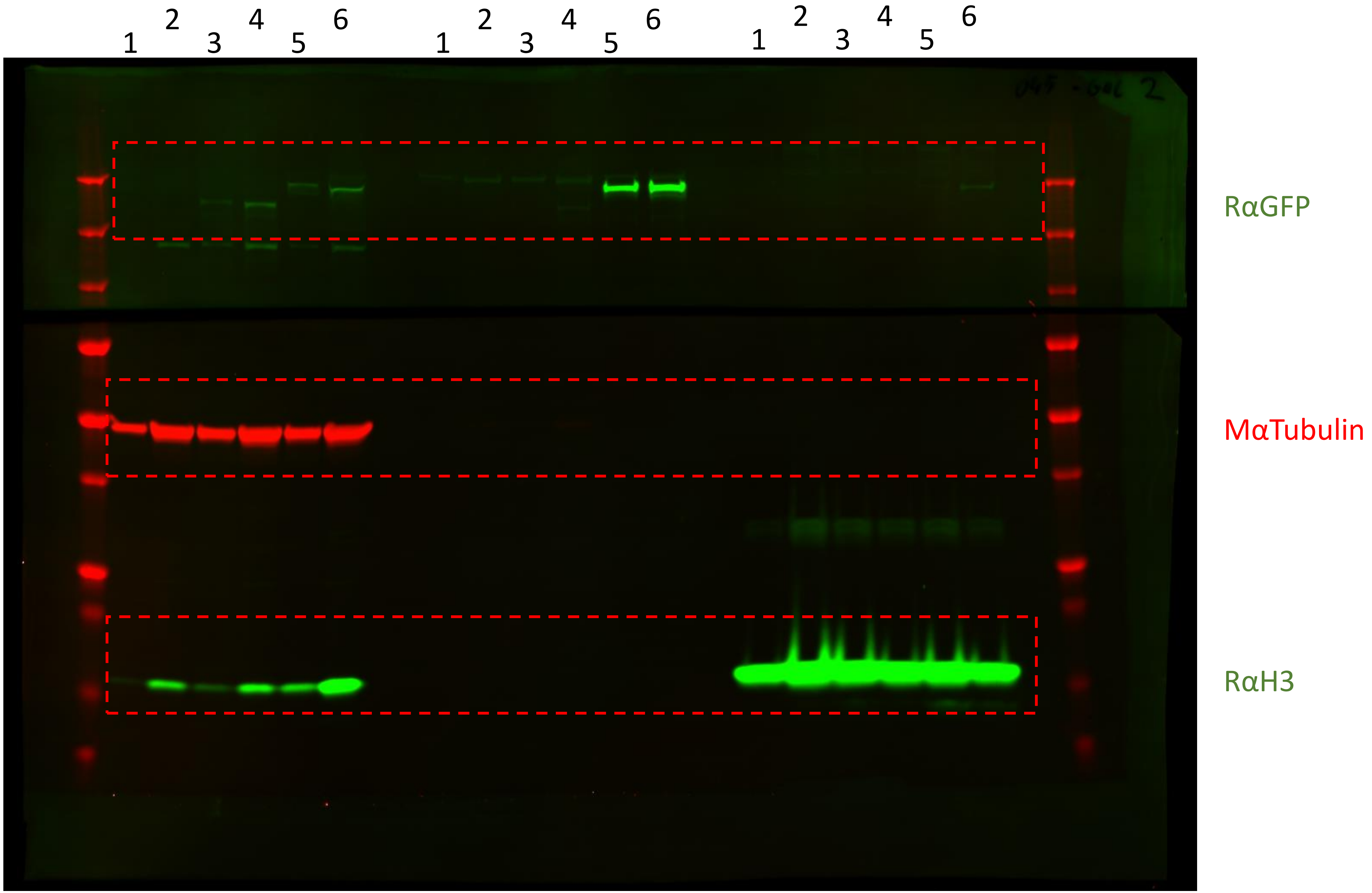

Goossens et. al.

Supp. Figure 1E

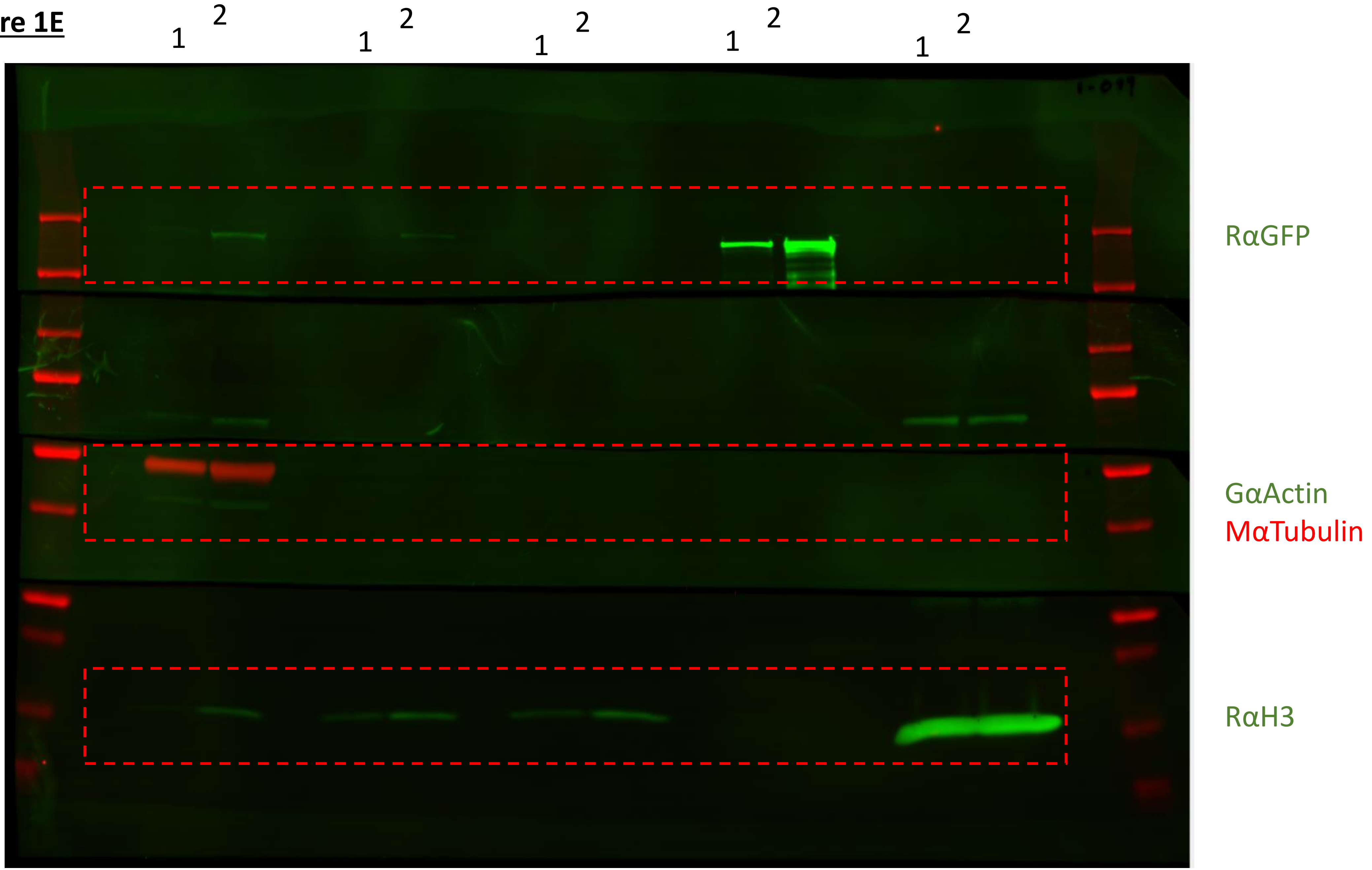

Goossens et. al.

Supp. Figure 3A

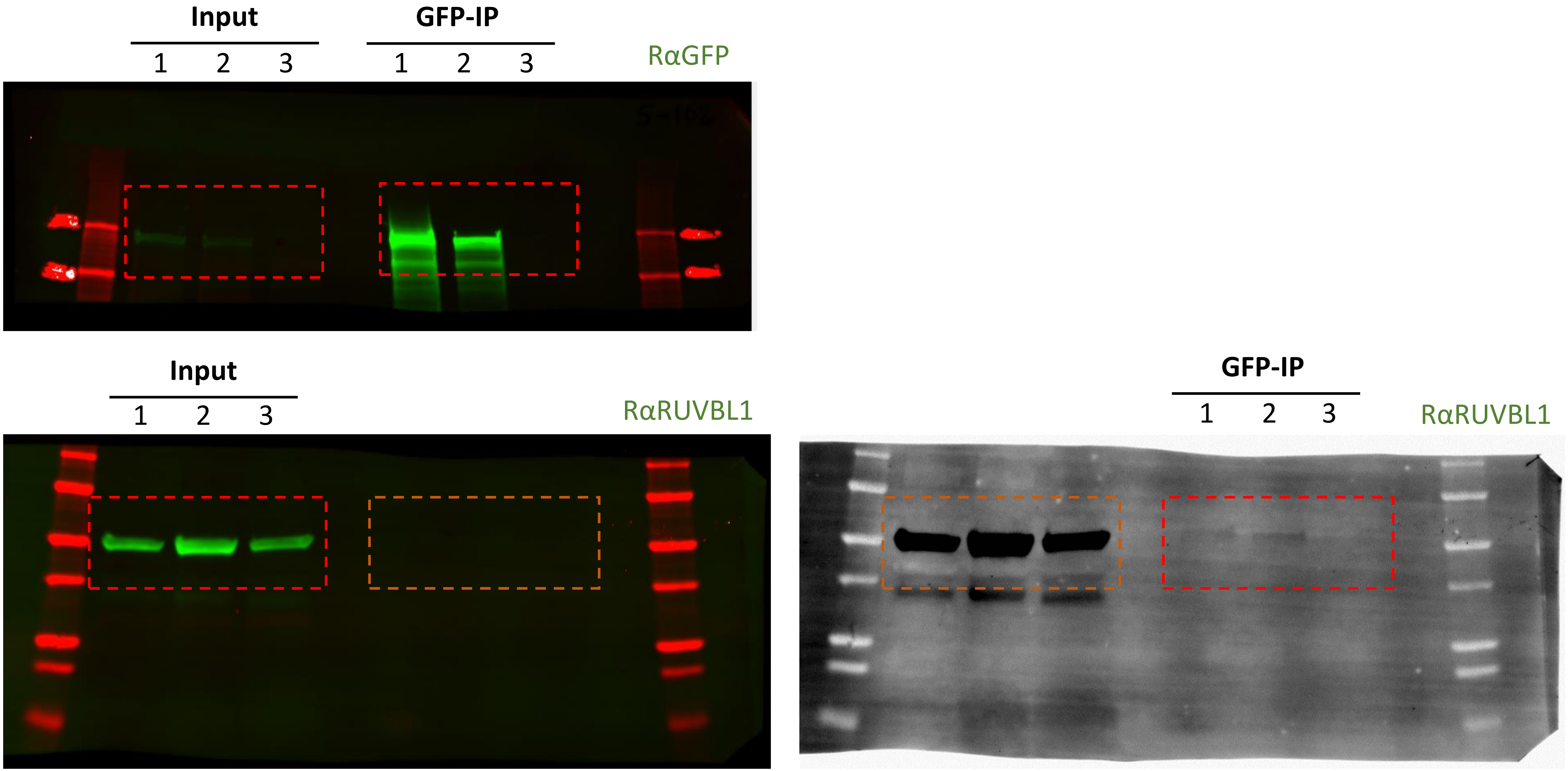

Low exposure

High exposure – channel 800 only

Goossens et. al.

Supp. Figure 3B (Input)

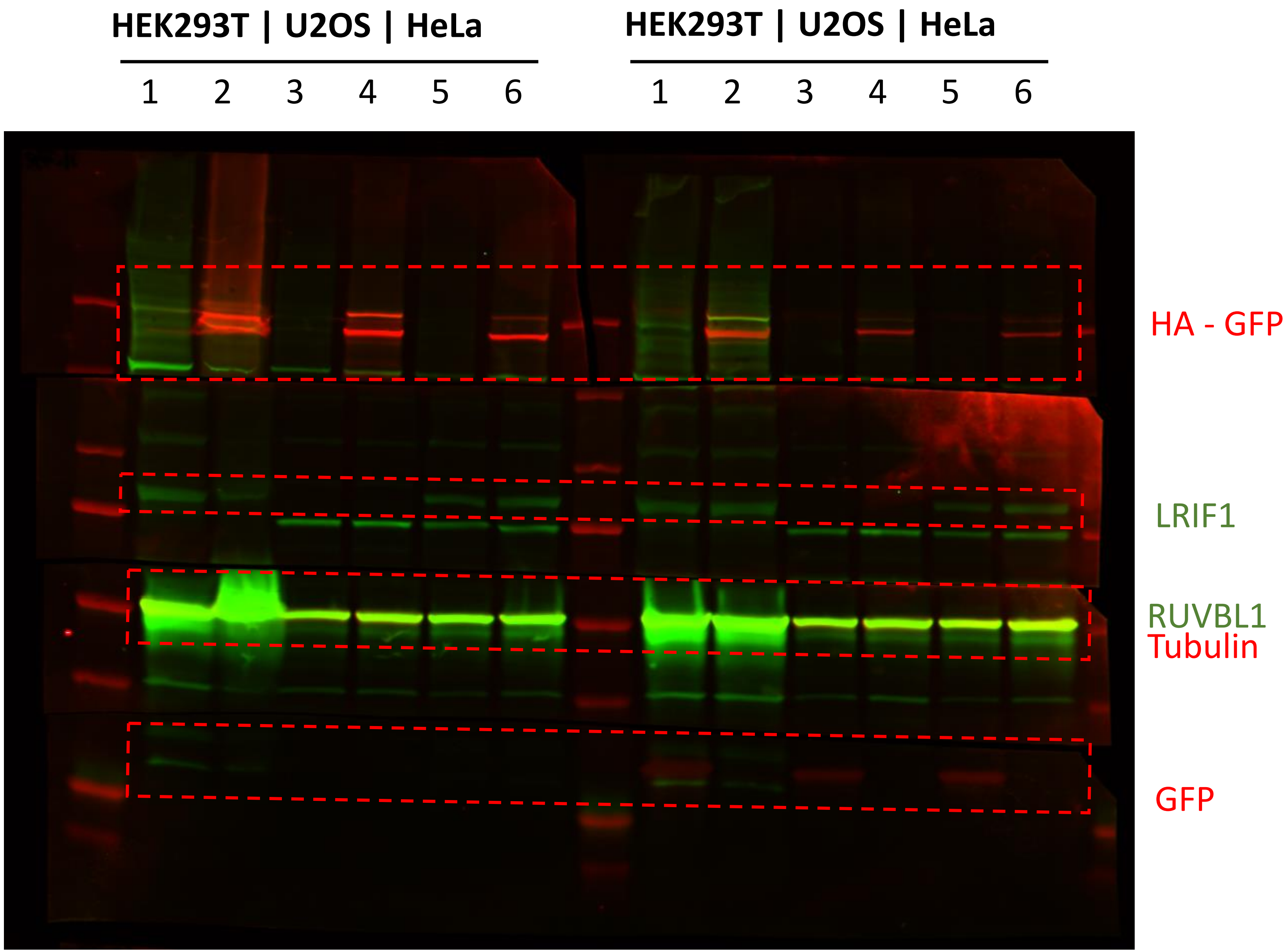

Goossens et. al.

Supp. Figure 3B (IP)

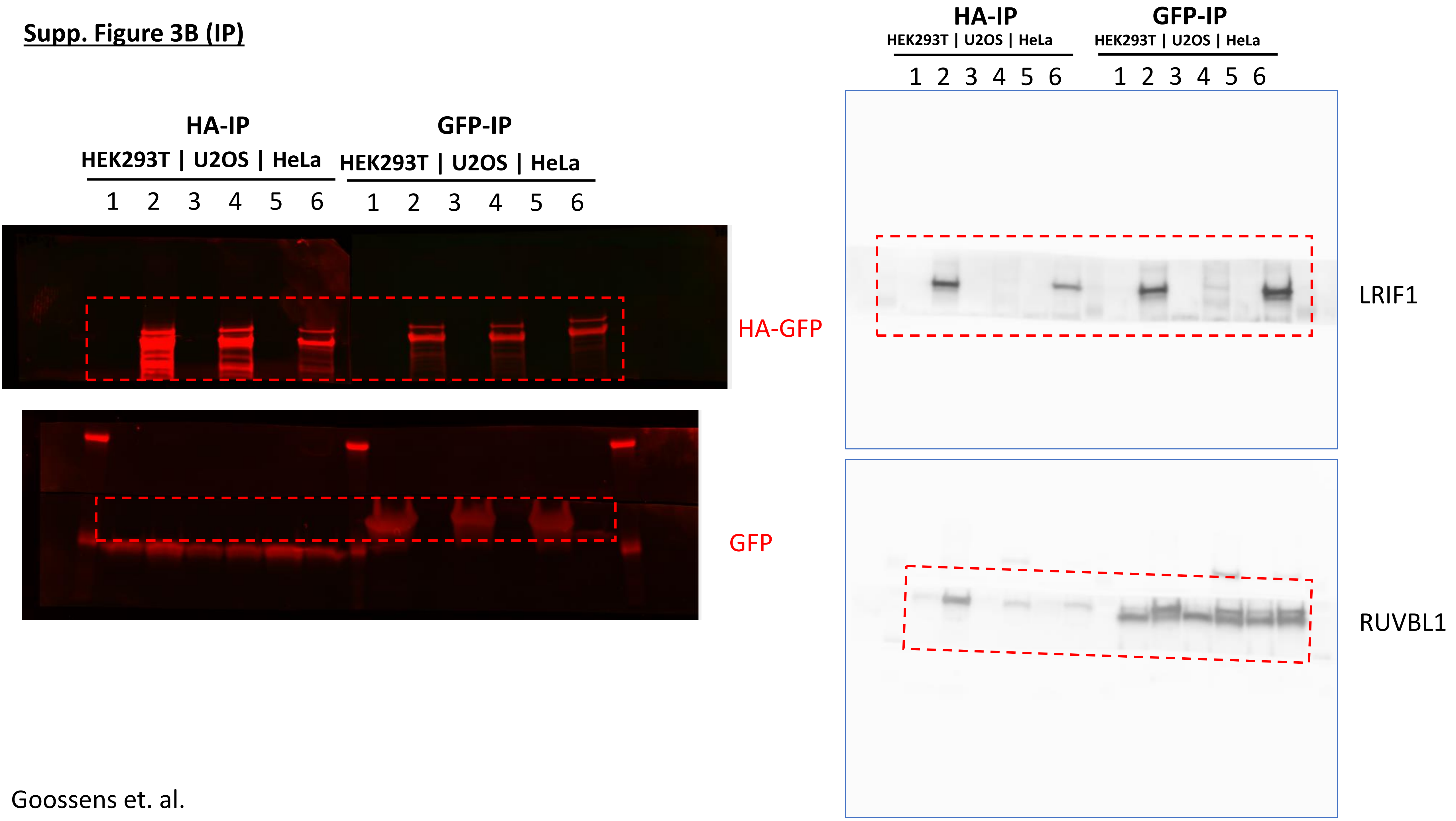

Goossens et. al.

Supp. Figure 3C

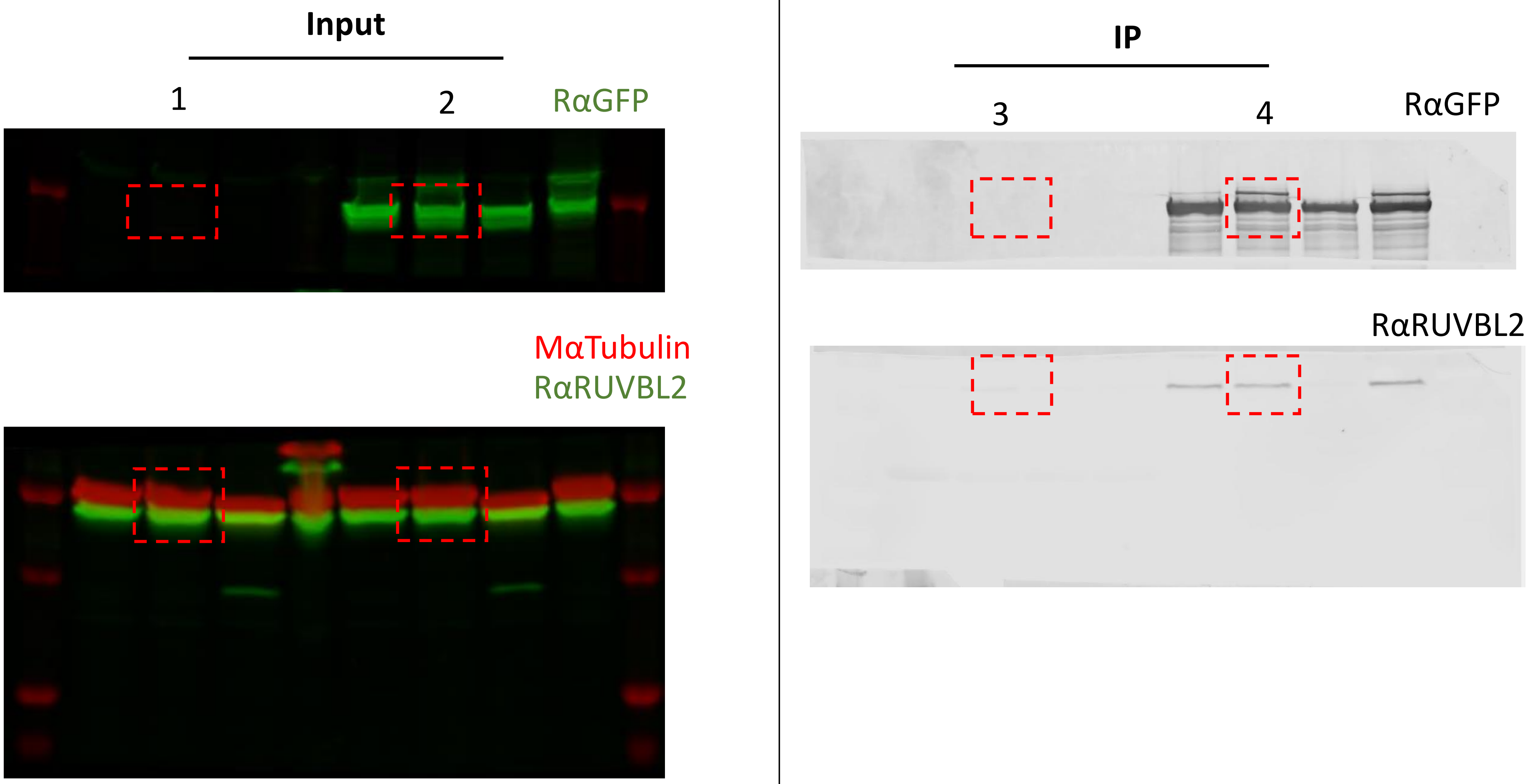

Goossens et. al.

Supp. Figure 3D

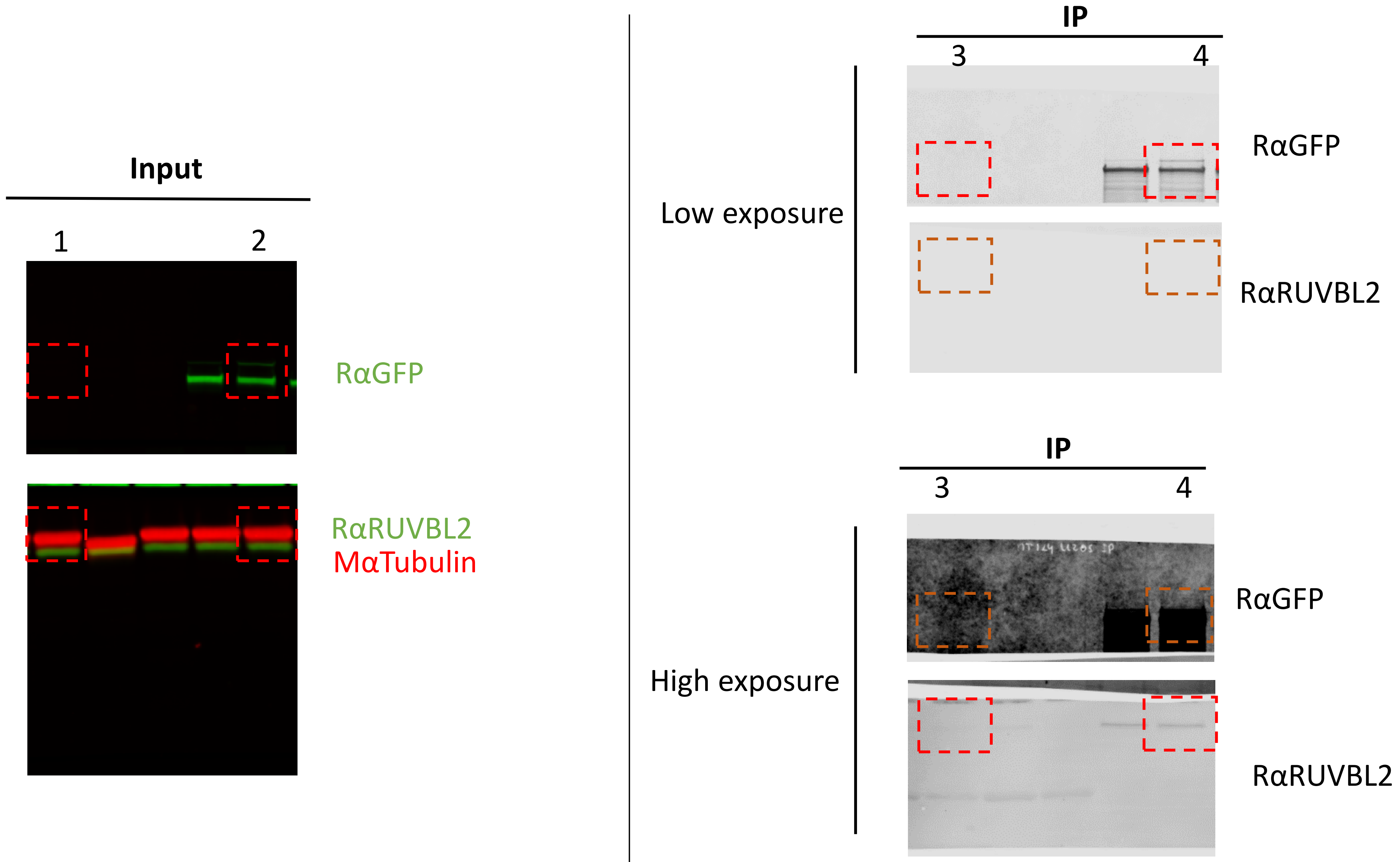

Goossens et. al.

Supp. Figure 3E

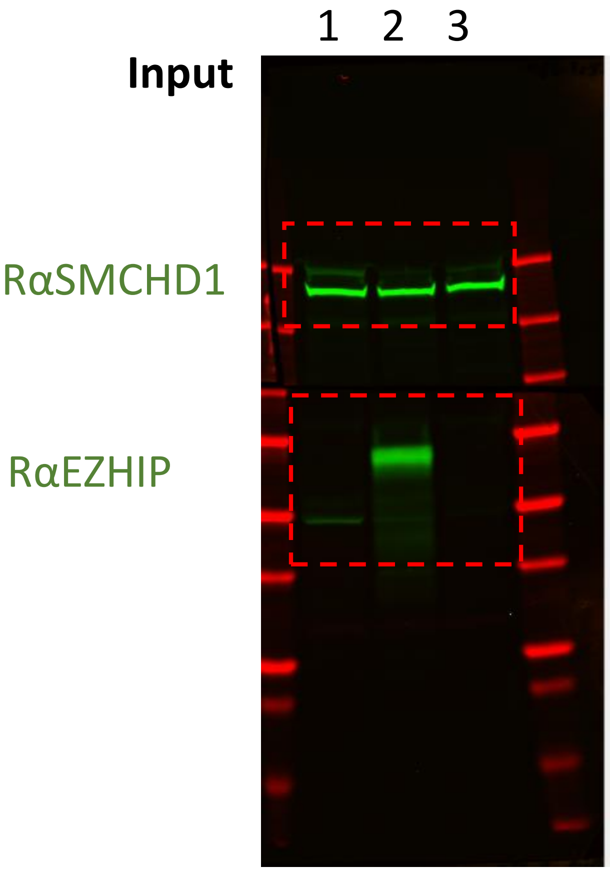

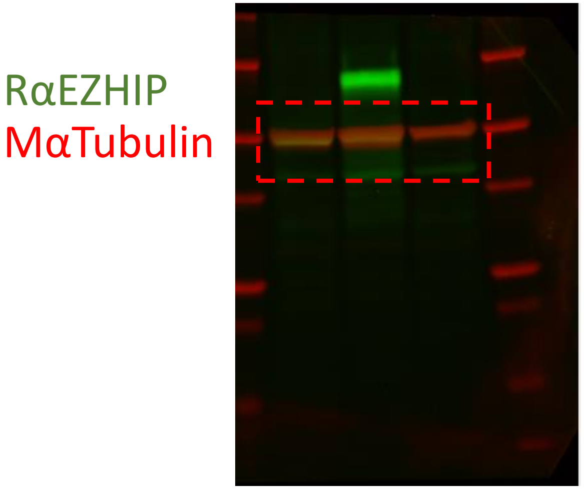

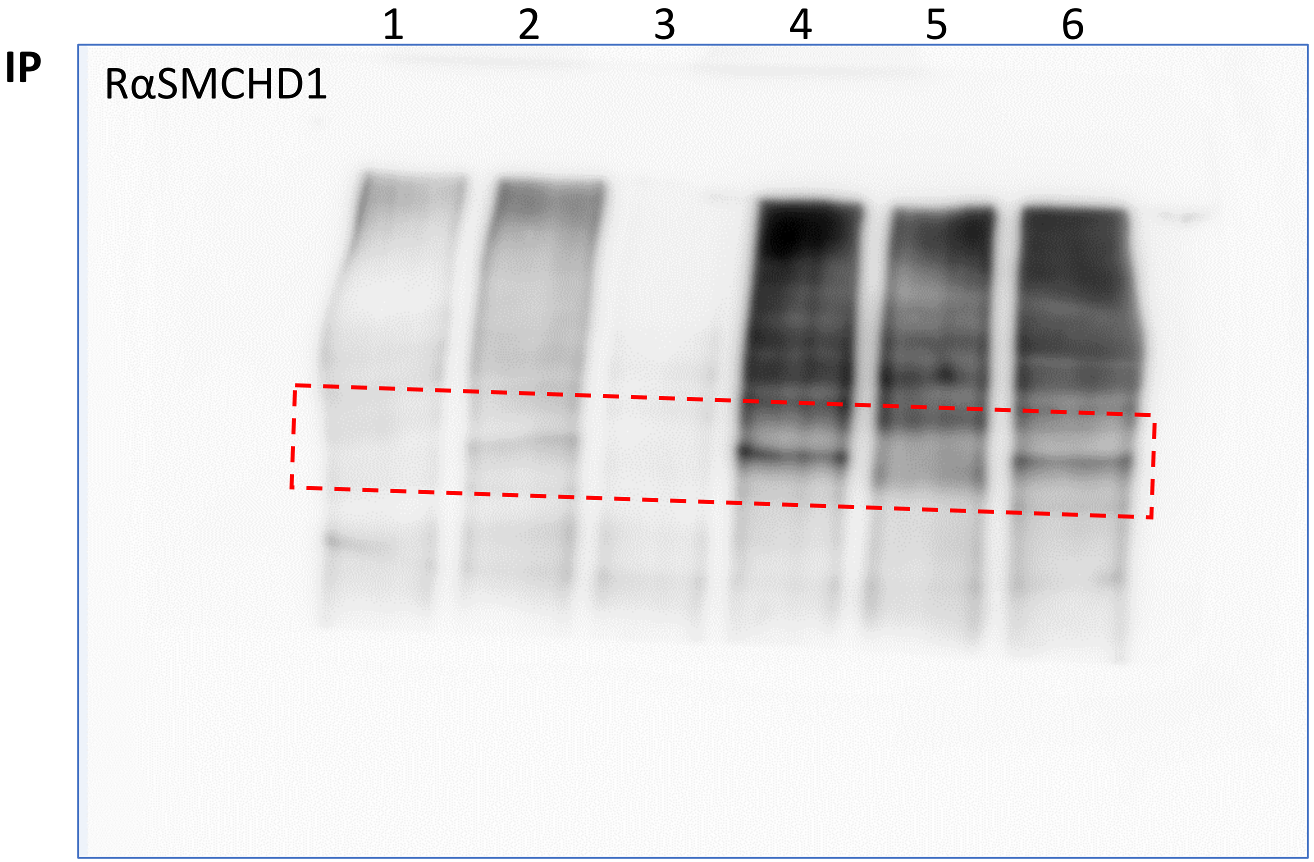

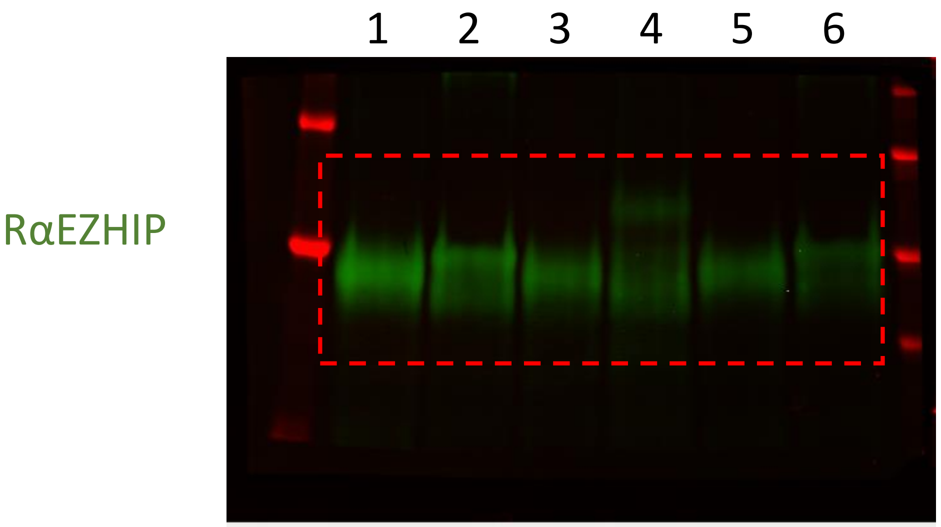

Goossens et. al.

Supp. Figure 4D

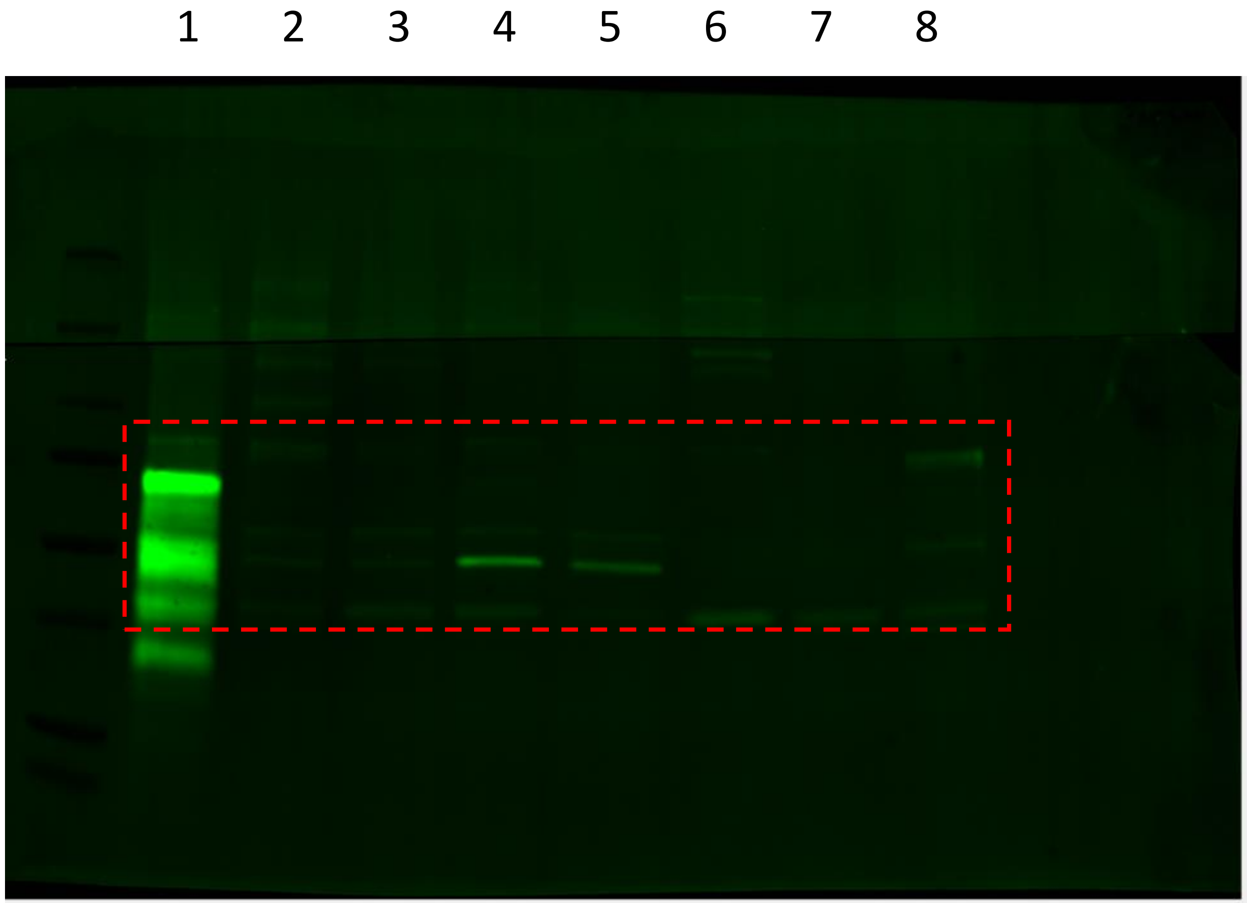

Channel 800nm
(RαEZHIP)

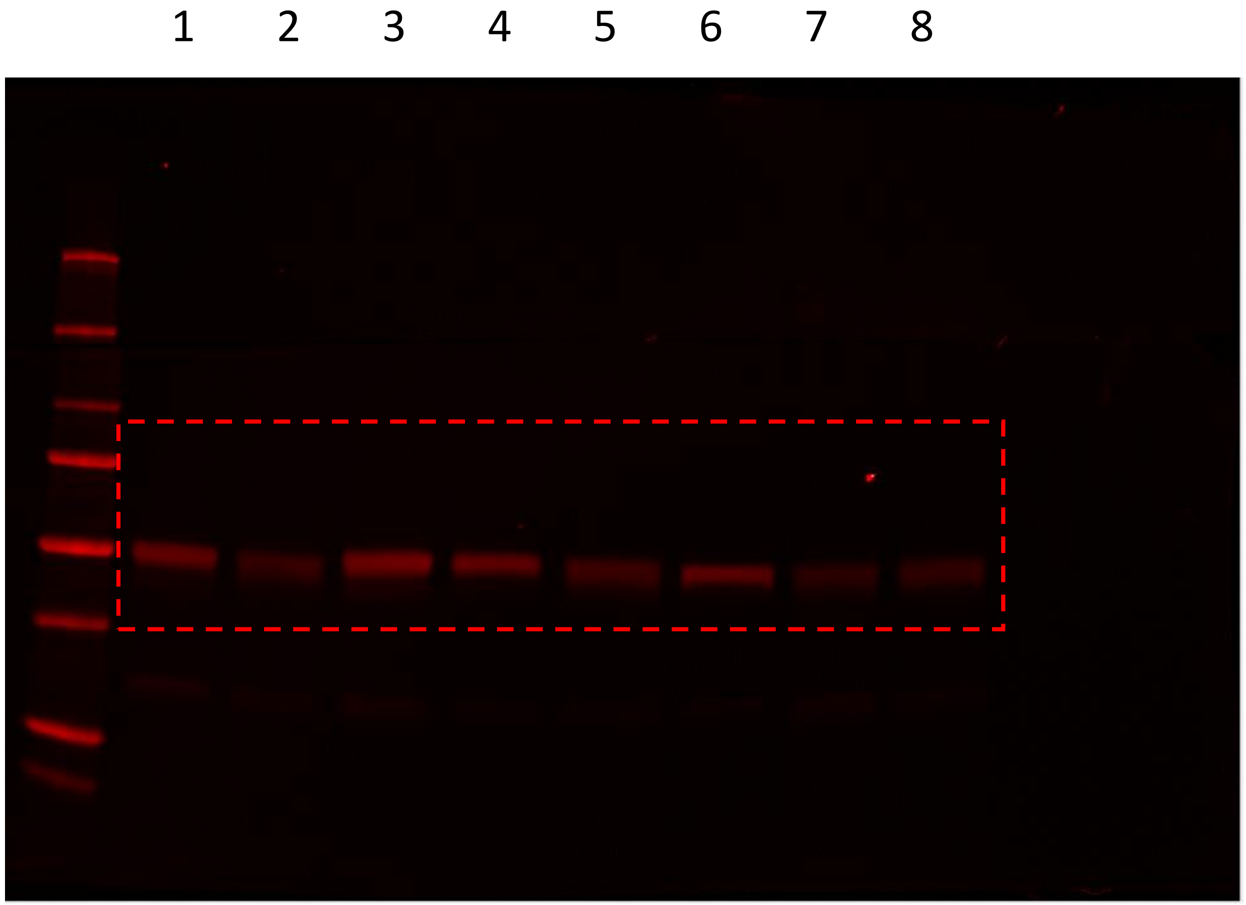

Channel 680nm
MαTubulin

Goossens et. al.

Supp. Figure 6B

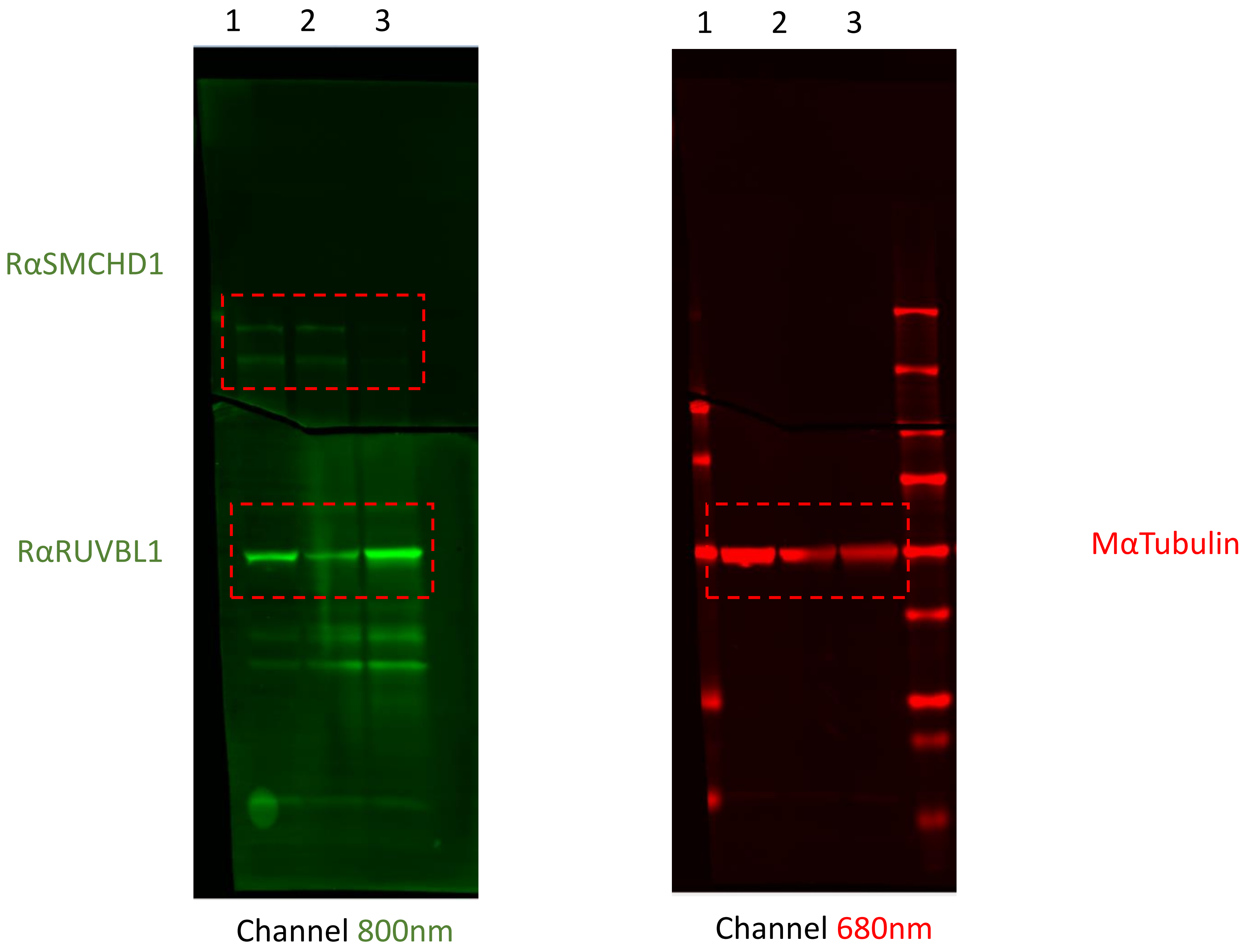

Goossens et. al.
